# Metabolomic profiles of metformin in breast cancer survivors: a pooled analysis of plasmas from two randomized placebo-controlled trials

**DOI:** 10.1186/s12967-022-03809-6

**Published:** 2022-12-29

**Authors:** Federica Bellerba, Anastasia Chrysovalantou Chatziioannou, Paniz Jasbi, Nivonirina Robinot, Pekka Keski-Rahkonen, Amarine Trolat, Béatrice Vozar, Sheri J. Hartman, Augustin Scalbert, Bernardo Bonanni, Harriet Johansson, Dorothy D. Sears, Sara Gandini

**Affiliations:** 1 Department of Experimental Oncology, IEO, European Institute of Oncology IRCCS, Milan, Italy; 2 International Agency for Research on Cancer, Nutrition and Metabolism Branch, Lyon, France; 3 College of Health Solutions, Arizona State University, Phoenix, AZ USA; 4 School of Molecular Sciences, Arizona State University, Tempe, AZ USA; 5 Herbert Wertheim School of Public Health and Human Longevity Science, UC San Diego, La Jolla, CA USA; 6 Moores Cancer Center, UC San Diego, La Jolla, CA USA; 7 Division of Cancer Prevention and Genetics, IEO, European Institute of Oncology IRCCS, Via Giuseppe Ripamonti 435, 20141 Milan, Italy; 8 Department of Medicine, UC San Diego, La Jolla, CA USA

**Keywords:** Cancer, Recurrence, Metabolic syndrome, Prevention, Lipids, Weight loss

## Abstract

**Background:**

Obesity is a major health concern for breast cancer survivors, being associated with high recurrence and reduced efficacy during cancer treatment. Metformin treatment is associated with reduced breast cancer incidence, recurrence and mortality. To better understand the underlying mechanisms through which metformin may reduce recurrence, we aimed to conduct metabolic profiling of overweight/obese breast cancer survivors before and after metformin treatment.

**Methods:**

Fasting plasma samples from 373 overweight or obese breast cancer survivors randomly assigned to metformin (n = 194) or placebo (n = 179) administration were collected at baseline, after 6 months (Reach For Health trial), and after 12 months (MetBreCS trial). Archival samples were concurrently analyzed using three complementary methods: untargeted LC–QTOF-MS metabolomics, targeted LC–MS metabolomics (AbsoluteIDQ p180, Biocrates), and gas chromatography phospholipid fatty acid assay. Multivariable linear regression models and family-wise error correction were used to identify metabolites that significantly changed after metformin treatment.

**Results:**

Participants (n = 352) with both baseline and study end point samples available were included in the analysis. After adjusting for confounders such as study center, age, body mass index and false discovery rate, we found that metformin treatment was significantly associated with decreased levels of citrulline, arginine, tyrosine, caffeine, paraxanthine, and theophylline, and increased levels of leucine, isoleucine, proline, 3-methyl-2-oxovalerate, 4-methyl-2-oxovalerate, alanine and indoxyl-sulphate. Long-chain unsaturated phosphatidylcholines (PC ae C36:4, PC ae C38:5, PC ae C36:5 and PC ae C38:6) were significantly decreased with the metformin treatment, as were phospholipid-derived long-chain n-6 fatty acids. The metabolomic profiles of metformin treatment suggest change in specific biochemical pathways known to impair cancer cell growth including activation of CYP1A2, alterations in fatty acid desaturase activity, and altered metabolism of specific amino acids, including impaired branched chain amino acid catabolism.

**Conclusions:**

Our results in overweight breast cancer survivors identify new metabolic effects of metformin treatment that may mechanistically contribute to reduced risk of recurrence in this population and reduced obesity-related cancer risk reported in observational studies.

**Trial registration:**

ClinicalTrials.gov identifier: NCT01302379 and EudraCT Protocol #: 2015-001001-14.

**Supplementary Information:**

The online version contains supplementary material available at 10.1186/s12967-022-03809-6.

## Background

Female breast cancer is currently the most diagnosed cancer in many countries worldwide [1]. Survivors of breast cancer are the largest population of cancer survivors, numbering more than 3.8 million women as of January 2019 in the United States alone [2] with > 90% survival after 5 years [3]. Cancer recurrence is a major health concern in this population, particularly in those with overweight or obesity [4]. Interventions that reduce recurrence risk are needed. In addition, analyses of underlying molecular mechanisms associated with recurrence-reducing interventions can provide insight into biochemical pathways that mediate cancer risk.

Metformin is the most widely prescribed medication to improve glycemic control in individuals with type 2 diabetes. In addition to its glucose lowering effects, metformin use is associated with clinically significant weight loss and improved insulin sensitivity [5]. Epidemiological studies show that metformin use diminishes cancer occurrence, suggesting that metformin intervention may reduce risk of recurrence in survivors of obesity-related cancers, e.g., breast cancer [6].

Molecular mechanisms that mediate the metabolic benefits of metformin include inhibition of gluconeogenesis (hepatic and renal) [7], activation of AMP-activated protein kinase (AMPK) [8], and inhibition of mitochondrial respiration and glycerophosphate dehydrogenase [9]. Recent studies demonstrate that additional metabolically beneficial effects of metformin are mediated by the gut, including alterations in enterocytes and microbiota [10]. However, the mechanisms by which metformin improves metabolic and cancer outcomes are not yet fully understood.

In this study, we employed targeted and untargeted metabolomics approaches to explore metabolites and metabolic pathways associated with metformin treatment in breast cancer survivors. To enhance statistical power, we conducted metabolomic profiling of metformin treatment in plasma samples at baseline and follow-up from two randomized controlled trials, testing the impact of metformin on body weight and the metabolic profile among 373 breast cancer survivors, the Reach for Health Study (US-based) and the MetBreCS study (Italy-based).

## Materials and methods

### Study design

This pooled analysis includes participants (*n* = 373) from two different randomized, double-blind, placebo-controlled trials enrolling overweight and obese breast cancer survivors with localized breast cancer disease at diagnosis.

**Fig. 1:**
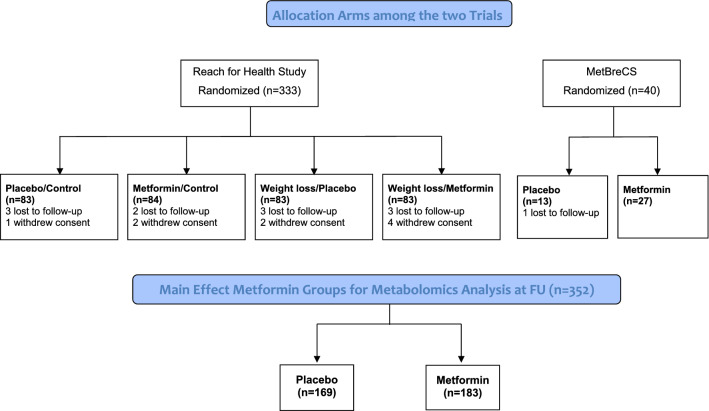
Flow diagram showing study design of the two trials and metformin main effect comparison groups for pooled metabolomic analysis

The Reach for Health Study (RFH) was approved by the Human Research Protections Program at UC San Diego, and participants signed informed consent forms (ClinicalTrials.gov identifier: NCT01302379; https://clinicaltrials.gov/ct2/show/NCT01302379). Details regarding the study design, recruitment strategies, interventions and primary outcomes have been previously published [11, 12]. Briefly, overweight/obese postmenopausal breast cancer survivors (n = 333, BMI ≥ 25.0 kg/m^2^) were randomly assigned to a 6-month treatment with metformin versus placebo and, in addition, assigned to a weight loss intervention versus control in a 2 × 2 factorial fashion (Fig. 1a). Fasting blood specimens and relevant clinical data were collected at baseline and at the final 6-month visit, with the termination of the treatment. Participants with self-reported diabetes were excluded from the study unless it was controlled solely with diet and lifestyle. Participants receiving hormone replacement therapy and/or having other serious medical conditions were ineligible.

The MetBreCS trial was a mono-institutional, randomized placebo-controlled phase II study of metformin treatment in breast cancer survivors at higher risk of recurrence (TNBC, non-luminal HER2+, and Luminal B HER2+) with BMI ≥ 25.0 kg/m^2^. Participants were excluded if they had diabetes or were taking metformin. Information regarding concomitant medications was collected, and none of the participants in the MetBreCS trial took insulin lowering drugs. MetBreCS was conducted at Milan-Italy, and included overweight/obese women having completed their adjuvant therapy (EudraCT Number: 2015-001001-14; https://www.clinicaltrialsregister.eu/ctr-search/search?query=2015-001001-14) (n = 40, BMI ≥ 25.0 kg/m^2^). The trial was approved by the local IRB and participants signed informed consent. Fasting blood specimens were collected at baseline and at 12 months.

All participants were randomly assigned to the metformin or to the placebo group, and both participants and study staff remained blinded to the medication group. Drug dose was gradually increased, to eliminate potential gastrointestinal side effects, starting from one 500 mg metformin (or placebo) tablet to 2 tablets/day after a week and 3 tablets/day after a month for the RFH cohort, and from a 850 mg tablet of metformin or placebo for the first 3 days to 2 tablets of 850 mg for the 1-year period for the MetBreCS study. By the end of the period of the trial, any unused medication was returned to the clinic, providing information on the medication adherence.

The pre-analytical process was optimized to avoid variability. Plasma EDTA morning fasting samples were separated and stored at − 80 °C. No thaw and freezing cycles were performed before analyzing the samples. Paired baseline and endpoint samples were analyzed consecutively and in random order, and the pairs were analyzed in randomized order across the batches. The batches included similar proportions of metformin and placebo group samples as well as RFH and MetBreCS samples in each batch.

### Untargeted metabolomics analyses

#### Sample preparation

The 725 EDTA plasma samples (baseline n = 373, follow-up n = 352) were prepared by mixing 30 *μ*L of plasma with 200 *μ*L of cold acetonitrile (CHROMASOLV LC–MS Ultra, Honeywell) for protein precipitation. The mix was centrifuged at 500*xg* (10 min, at 4 °C), and the precipitate was filtered with 0.2 μm ND Captiva filter plates (Agilent Technologies). One hundred microliter of the filtrate was mixed with an equal volume of ultrapure water (18.2 MΩ cm, 1 ppb, Thermo Scientific) in Agilent 96-well plates, then sealed (BioChromato Rapid EPS, Fujisawa, Japan), and analyzed immediately. Quality control (QC) and blank samples were also prepared and analyzed along and in the same manner as the study samples. The former derived from 79 randomly selected and pooled study plasma samples, whereas the latter consisted of only acetonitrile. Each well plate included four individually prepared QCs and two blanks.

#### Sample analysis

Sample extracts were split into two independent analytical batches of four 96-well plates each. Samples were kept at 4 °C and 2 µL was injected to a tandem ultra-high-performance liquid chromatography–quadrupole time-of-flight mass spectrometry system (UHPLC-QTOF-MS, Agilent 1290 Infinity Binary LC system and 6550 QTOF mass spectrometer with Jet Stream electrospray ionization source, controlled by MassHunter Acquisition 10.1 software of Agilent Technologies). The samples were separated through a reversed phase column (ACQUITY UHPLC HSS T3, 2.1 × 100 mm, 1.8 μm, Waters), set at 45 °C, using two mobile phases: ultrapure water (as described earlier) and LC–MS grade methanol (CHROMASOLV LC–MS Ultra, Honeywell), both containing 0.05% (v/v) of formic acid. Additional method details have been described earlier [13].

#### Data processing

Pre-processing was performed using Profinder 10.0 and Mass Profiler Professional B.14.9.1 software (Agilent Technologies). A “Batch recursive feature extraction (small molecules)” process was employed to find [M + H]^+^ ions. Height thresholds of 1500 and 8000 counts for mass and chromatographic peaks were used, respectively, and a minimum quality score of 70. Feature alignment between samples was performed with retention time and mass windows of ± 0.05 min and ± (15 ppm + 2 mDa), respectively. A target list for the recursive extraction was created by including features fulfilling the above criteria in at least 20 samples. Recursive feature extraction was then performed using ± 25 ppm m/z width to draw chromatographic peaks, Agile 2 integrator without smoothing, and the mass was calculated as an average from spectra > 80% peak height. Matching tolerances for retention time and mass were ± 0.05 min and ± 10 ppm, respectively. The resulting data was exported as a.pfa file into Mass Profiler Professional and features present in every blank were excluded, unless fivefold greater in average intensity in samples within the same analytical batch. Peak areas were used as a measurement of intensity.

#### Statistical methods

Thirty-six negative intensities (0.002% of all features intensities) were found and replaced by “0” whenever they occurred. The filtration based on missing values was conducted per group (placebo and metformin) and features with less than 20% of missing values at both time points were retained, to avoid substantial imputation. A total of 777 and 778 features out of all 2069 were retained in the placebo and metformin arms respectively, and a total of 755 features for the pooled analysis (RFH + MetBreCS).

The intensities of the retained features were log-transformed and imputed using a quantile regression approach for left-censored missing data (‘imputeLCMD’ R package). After imputation, all features were brought back to the original scale. Principal component analysis (PCA) based on covariance matrix was performed on the pooled sample including the changes from baseline of the 755 features. The scaled scores of the first two components were then plotted to graphically assess heterogeneity related to the study center, and to investigate the presence of batch effects.

The assumption of Gaussian distribution was assessed by Shapiro–Wilk test for the raw values of the intensities and for the log-transformed values, as well as for the changes from baseline. In univariate analysis, change in the intensity of each feature was examined within each treatment group (baseline vs. final value). As only about 20% of the feature changes were normally distributed, the non-parametric Wilcoxon rank-sum test was used. All *P*-values were then adjusted for false discovery rate (FDR) estimation through the Benjamini–Hochberg correction, and only FDR-corrected *P*-values < 0.05 were considered statistically significant.

**Fig. 2:**
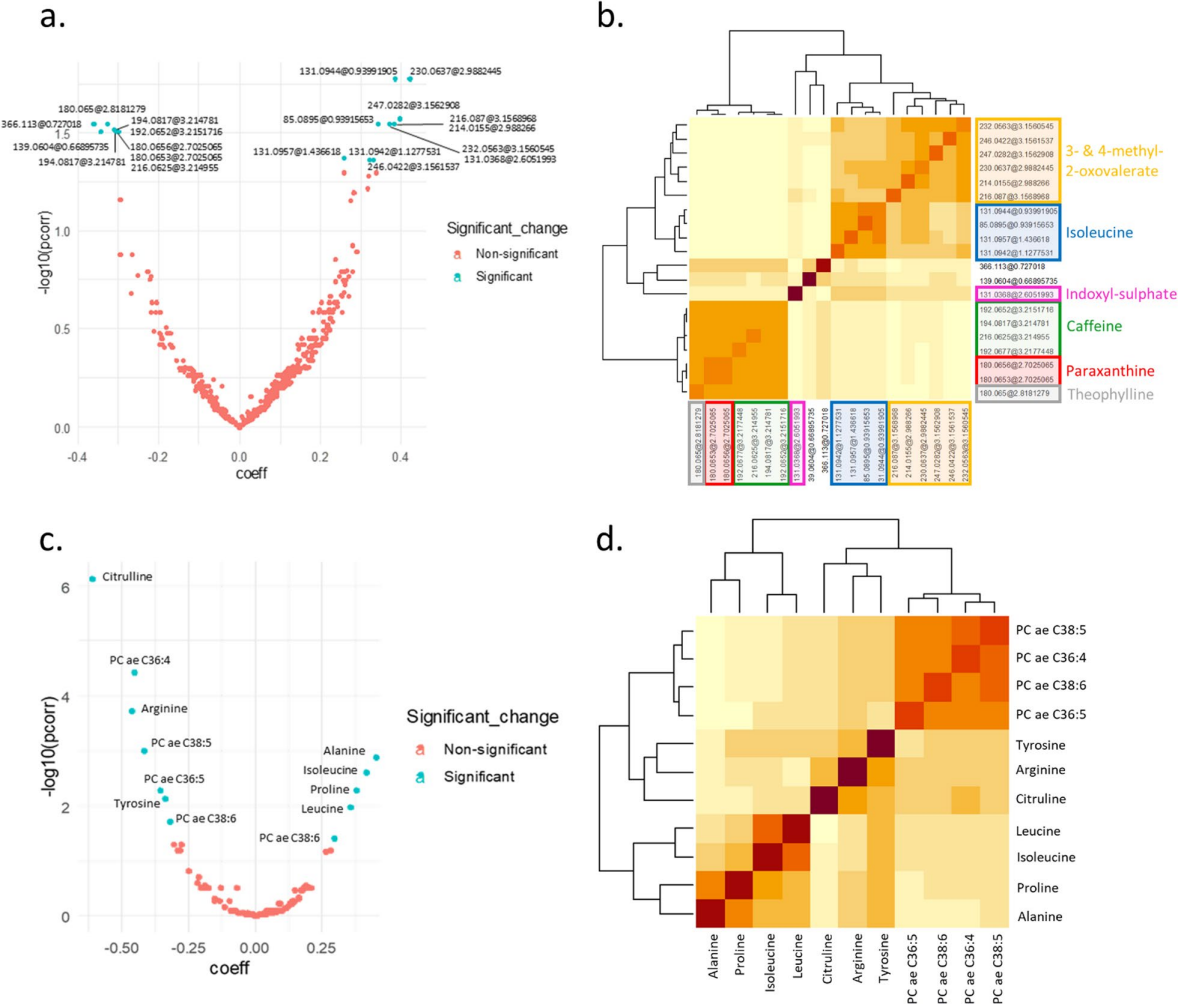
Volcano plots of detected metabolomic features and relative Spearman-based heatmap of the significant feature changes, applying untargeted and targeted metabolomics. **a**, **c** Volcano plots of detected features from untargeted (**a**) and targeted (**c**) metabolomics analyses. The beta regression coefficients of the treatment effect are plotted in the x-axis, and the − 10log (FDR-corrected *P*-values) in the y-axis. *P*-values were corrected using the Benjamini–Hochberg FDR method, considering a threshold of 0.05 for statistical significance. **b**, **d** Spearman-based heatmap of the significant feature changes detected from untargeted (**b**) and targeted (**d**) metabolomics analyses

Changes in the intensities of features were then compared between treatment groups (metformin vs. placebo) by using multivariable linear regression models on the pooled sample. Models were fit on the scaled feature changes and adjusted for the scaled baseline feature evaluation, weight-loss intervention, study center, age, change in body mass index (BMI), ongoing aromatase-inhibitor therapy and any tumor-related characteristics that were significantly unbalanced between the two study centers. The normal distribution of residuals from fully adjusted models was inspected visually. Results from these multivariate analyses were then graphically represented through a volcano plot, which included the beta regression coefficients of the treatment effect in the x-axis, and the −log_10_ (FDR-corrected *p*) in the y-axis (Fig. 2a, c). Because the weight loss could also be a consequence of metformin treatment, we performed sensitivity analyses controlling for baseline BMI rather than BMI change, to investigate if the change in weight confounded the associations between metformin and the feature changes found in the main analysis. We also carried out subgroup analyses in order to investigate the influence of study design/center. We evaluated whether the estimates of the effect of treatment on metabolites changes found in the pooled sample were consistent with the estimates found in the single studies. Bar plots of the estimates in each study are presented in Additional file 5: Fig. S5.

Heatmaps based on Spearman’s correlation were created for the significant feature changes found in either univariate or multivariate analysis, to graphically assess their correlation structure and identify clusters (Fig. 2b, d). Correlations were also investigated by computing partial correlation networks with the graphical LASSO algorithm [14], which included the feature changes and BMI change and used the extended Bayesian information criterion (EBIC) to select model complexity. This approach provided correlation estimates between each pair of feature changes, that were adjusted for all other remaining feature changes and BMI change.

All statistical analyses were performed using R Statistical Software, version 4.1.2. Additionally, pathway analysis was conducted using the MetaboAnalyst 5.0 online tool (www.metaboanalyst.ca) and KEGG library.

#### Identification of metabolites

The features indicated by the statistical analysis as significantly deviating between the placebo and the metformin arm were grouped by retention time and intensity correlation (Spearman) across all samples. In this way, features that most likely correspond to the same metabolite were identified and the most abundant features were selected to facilitate the annotation. Those features were first compared with the in-house database of analytical standards with 10 ppm molecular weight and 0.35 min retention time tolerance, considering [M + H]^+^ and [M + Na]^+^ adducts. A further search of the *m/z* values that did not match metabolites from the in-house library was conducted against the Human Metabolome Database (HMDB) [15] and using MyCompoundID [16].

The best matching identities were confirmed by re-analysis of the sample with the highest intensity of the corresponding feature together with the analytical standard, allowing the confirmation of the exact retention time and shape of the peak. MS/MS spectra were also collected for the sample and the analytical standard, allowing the confirmation of the identification of the metabolite to confidence level 1 [17].

### Targeted metabolomics analyses

#### Sample preparation and analysis

A standardized protocol for sample processing was followed. All plasma samples were assayed at IARC, using the AbsoluteIDQ p180 Kit (Biocrates Life Sciences AG, Innsbruck, Austria). Same principles were applied for sample randomization as for untargeted metabolomics, and the 725 samples were analyzed in 10 individual batches along with quality control samples from pooled plasma. Laboratory personnel were blinded to sample categories, that is, before and after treatment or placebo and metformin groups. A triple quadrupole mass spectrometer (Triple Quad 4500, AB Sciex, Framingham, MA) was used to quantify a total of 145 metabolites. Details for the quantified targets/metabolites and their chemical classes are provided in Additional file 6: Table S1. Some metabolites were excluded due to missing data, i.e., values outside range for quantification or coefficient of variation higher than 20% in the QC samples.

#### Statistical methods

For each metabolite, missing values were replaced with the limit of detection (LOD) or with the lower limit of quantification (LLOQ), depending on the availability of the limit per metabolite. Two metabolites, namely spermine and spermidine, were excluded from further analysis, as they were missing for more than 30% of the sample. Statistical analysis was the same as described in the *Untargeted metabolomics* section.

### Fatty acid analyses

#### Sample preparation and analysis

Plasma samples were profiled for phospholipid fatty acid composition in batches of twenty. Samples were randomized as explained above and two independent samples were used as quality controls. Total lipids were extracted from samples, phospholipids purified by adsorption chromatography, fatty acids trans-esterified and fatty acid methyl esters quantified by gas chromatography as previously described [18]. The relative amount of each fatty acid, expressed as a percentage of total fatty acids, was determined by integrating the area under the curve for each fatty acid and dividing by the total area.

#### Statistical methods

A total of 61 fatty acid categories were considered in the analysis: 40 individual fatty acids and 21 fatty acid groups calculated from the individual ones. Details for the quantified fatty acids and the groups of fatty acids are provided in Additional file 7: Table S2. One participant had a missing value of the ratio 18:3n − 6/18:2n − 6 at follow-up, which was imputed with the value of the same fatty acid at baseline. Percentage of 20:0 and percentage of 24:0 over the total fatty acids content were zero for all participants at both time points, so they were excluded from the analysis. To take into account the compositional nature of the data, we evaluated the change in time of each fatty acid as following: if the fatty acid increased from baseline, considering as reference the median values, then it was classified as “increase”, if the fatty acid decreased from baseline then it was classified as “decrease”, it was classified as “stable” in all the other cases.

For the 40 individual fatty acids, the Least Absolute Shrinkage and Selection Operator (LASSO) [19] logistic regression model was implemented to select the fatty acids associated with the treatment. The best lambda parameter was estimated using leave-one-out cross-validation, while the regularization strength was selected as the minimum value that maximized the deviance of the model.

All the selected fatty acids were eventually used as covariates in a multivariate logistic regression model to investigate the association between metformin and increasing/decreasing levels of fatty acids, considering the “stable” classification described above as reference. Estimates of the significant associations between fatty acids and study treatment were provided as Odds Ratios (ORs) and 95% confidence intervals (95%CI). The model was adjusted for the same factors described in the Untargeted metabolomics section.

The same approach was used separately for the 21 composite fatty acid groups.

As for metabolomics analysis, sensitivity analyses controlling for baseline BMI instead of BMI change were run to verify whether the effect of metformin on weight change confounded the strength of the associations between metformin and fatty acid increases/decreases identified in the main analysis.

## Results

### Participant demographics

A total of 373 breast cancer survivors were randomized in the two original studies. Of those, 352 participants had both baseline and follow-up plasma samples available. Participant allocations to study arms across studies and in the combined analysis are presented in Fig. 1. Participant characteristics for the MetBreCS and RFH studies are shown in Additional file 8: Table S3 and summarized by treatment group for the combined sample in Table 1.

**Table 1:** Participant baseline characteristics by treatment group in the pooled sample (ITA + USA)

	Placebo (N = 179)	Metformin (N = 194)	*P*-value^a^
Weight loss intervention n (%)			
No	96 (53.6%)	111 (57.2%)	0.554
Yes	83 (46.4%)	83 (42.8%)	
Age, median (Q1, Q3)	63.0 (57.0, 67.0)	60.0 (56.0, 66.0)	0.031
BMI, median (Q1, Q3)	29.8 (27.5, 33.1)	30.0 (27.4, 33.4)	0.889
Menopausal status, n (%)			
Post-menopausal	179 (100%)	183 (94.3%)	0.003
Pre-menopausal	0 (0%)	11 (5.7%)	
Pathologic stage, n (%)			
Stage I	85 (47.5%)	92 (47.4%)	0.986
Stage II	63 (35.2%)	68 (35.1%)	
Stage III	31 (17.3%)	32 (16.5%)	
Missing	0 (0%)	2 (1.0%)	
Histology, n (%)			
Invasive ductal carcinoma	130 (72.6%)	154 (79.4%)	0.310
Invasive lobular carcinoma	21 (11.7%)	17 (8.8%)	
Other/unknown	28 (15.6%)	23 (11.9%)	
Tumor grade, n (%)			
Grade I	46 (25.7%)	49 (25.3%)	0.972
Grade II	75 (41.9%)	78 (40.2%)	
Grade III	53 (29.6%)	62 (32.0%)	
Unknown	5 (2.8%)	5 (2.6%)	
Estrogen receptor status, n (%)			
Negative	32 (17.9%)	51 (26.3%)	0.072
Positive	146 (81.6%)	143 (73.7%)	
Missing	1 (0.6%)	0 (0%)	
Progesterone receptor status, n (%)			
Negative	45 (25.1%)	67 (34.5%)	
Positive	126 (70.4%)	118 (60.8%)	
Borderline/not noted	7 (3.9%)	9 (4.6%)	0.125
Missing	1 (0.6%)	0 (0%)	
HER2 status, n (%)			
Negative	143 (79.9%)	139 (71.6%)	0.341
Positive	31 (17.3%)	44 (22.7%)	
Not noted/other	4 (2.2%)	5 (2.6%)	
Missing	1 (0.6%)	6 (3.1%)	
Aromatase inhibitor therapy, n (%)			
No	84 (46.9%)	99 (51.0%)	0.491
Yes	95 (53.1%)	95 (49.0%)	

^a^*P*-values derived from Wilcoxon rank-sum test for numerical variables and from Chi-square test (or Fisher exact test, where appropriate) for categorical variables

The two cohorts had significantly different baseline characteristics, with the Italian cohort being younger and with a lower BMI. Differences in menopausal status were observed, as the US study only included post-menopausal women and the Italian cohort included 11 pre-menopausal women. A great proportion of US study women (57%) were taking aromatase inhibitors. Differences in tumor histology, grade and stage at diagnosis were also observed, as well as in the status of the estrogen receptor, the progesterone receptor and HER2 (Additional file 8: Table S3). Thus, all these baseline imbalances were adjusted for in multivariate analyses. After pooling the two samples, no significant differences between placebo group and metformin group were found at baseline, with the exception of age, as the women in placebo were slightly older than the women in metformin group (median age: 63 vs. 60 years, respectively; Table 1). At the end of the study, a significant difference between the metformin and the placebo group was observed in the weight loss achieved, with metformin group losing more weight than placebo [median BMI change (final-baseline): − 1.22 vs. − 0.42 kg/m^2^ for Metformin group and Placebo group, respectively; Wilcoxon test, *P*-value < 0.0001].

### Untargeted metabolomics analysis

In univariate analysis, MS feature intensities were compared before and after treatment. After FDR multiple-testing correction, a total of 165 features significantly changed in the metformin-treated arm whereas no significant difference was found in the placebo arm.

Results from the PCA on the changes between baseline and end point are provided in Additional file 1: Fig. S1 (top). The scaled PCA scores of the first two components plotted indicate that the distribution of the samples is not affected by study center. Together, the first two components explain 25% of the total variability in placebo group and 22% of the total variability in metformin group. It is worth noting that the distribution is not related to the batches into which the samples were organized during the analysis. Moreover, the PCA analysis confirms the lack of any variability due to the study site origin of the plasma samples (RFH or MetBreCS cohort).

The multivariate analysis comparing the feature changes between treatment arms and adjusted for confounders resulted in 20 features significantly differing between the two treatment arms (Additional file 9: Table S4). Seven metabolites, corresponding to 18 of these features, were unambiguously identified (Level 1) using pure analytical standards (Table 2, and Additional file 2: Fig. S2).

**Table 2:** Level 1 identified metabolites with metformin treatment-associated changes over time that differ significantly from placebo

Annotated compound	Mass	Retention time (min)	Beta regression coefficient^a^	FDR-corrected *P*-value^b^	Direction of change with treatment	Coefficient of variation in QC samples (n = 32, %)
Caffeine	194.0817	3.21	− 0.31	0.031	↓	6.27
Paraxanthine	180.0656	2.70	− 0.30	0.031	↓	5.44
Theophylline	180.065	2.82	− 0.33	0.029	↓	6.70
Isoleucine	131.0957	1.44	0.26	0.043	↑	17.77
3-Methyl-2-oxovalerate^c^	230.0637	2.99	0.42	0.017	↑	7.02
4-Methyl-2-oxovalerate^c^	232.0563	3.16	0.37	0.029	↑	8.77
Indoxyl sulphate ^c^	131.0368	2.61	0.37	0.029	↑	24.14

Compound identified at Level 1, using the corresponding analytical standard for confirmation of retention time and MS/MS fragmentation spectra^3^

^a^Positive beta coefficient indicates a bigger increase in time of the metabolite in the metformin arm compared to placebo arm, whereas negative coefficient indicates a bigger decrease of the metabolite in the metformin arm

^b^*P*-value of the treatment covariate (Metformin vs. Placebo) derived from a multivariate linear model fit on scaled metabolite changes (final evaluation—baseline), adjusted for the scaled baseline value of the metabolite, study center, weight-loss intervention, age, change in BMI, ongoing aromatase-inhibitor therapy, histology, tumor grade, stage, HER2, progesterone receptor, estrogen receptor

^c^Compound identified by analyzing the sample on negative mode

Of the 7 metabolites, 3 decreased in the metformin arm compared to the placebo arm, namely caffeine, paraxanthine and theophylline, whereas 4 metabolites increased in the metformin arm compared to the placebo arm (isoleucine, 3-methyl-2-oxovalerate, 4-methyl-2-oxovalerate and indoxyl sulfate) (Additional file 3: Fig. S3). These results were confirmed in sensitivity analysis, where the models were adjusted for baseline BMI instead of BMI change (data not shown). The subgroup analysis by study shows that treatment effects on the 20 untargeted features observed in the pooled sample are consistent, although some not statistically significant in the smaller Italian sample (Additional file 5: Fig. S5, panel b).

### Targeted metabolomics analysis

In univariate analysis, after FDR correction, 6 metabolites in the placebo group and 52 metabolites in the metformin group were found to significantly change between the two time points (end point—baseline). In Additional file 1: Fig. S1 (bottom), the results from PCA on the metabolite changes between baseline and end point are provided. The scaled first two components of the subset of placebo (left), and metformin arm (right) are projected and colored by the study center (MetBreCS and RFH). Together, the first two components explained about 35% of total variance of data (33% in placebo group, 34% in metformin group). Overall, the PCA plots indicate that the distribution of the samples is not affected by the study center, with the first component explaining about 25% of the total variance.

Considering the same confounders as for the statistical analysis performed with the untargeted data, and applying the same multivariate regression models as earlier, 11 metabolite changes were found to be significantly different between the two treatment arms after FDR multiple-testing correction. Those metabolites are summarized in Table 3, providing the beta regression coefficients of the treatment covariate and the corrected *P*-values. The same significant metabolites changes between treatment arms were also observed in sensitivity analysis after controlling for baseline BMI, with similar regression coefficients and analogous interpretation (data not shown). The subgroup analysis by study shows that treatment effects on the 11 targeted metabolite changes observed in the pooled sample are consistent, although some of not statistically significant (Additional file 5: Fig. S5, panel a).

**Table 3:** Metformin treatment-associated metabolite changes over time that differ significantly from placebo in targeted analysis

Metabolite (scaled)	Beta regression coefficient^a^	FDR-corrected *P*-value^b^	Direction of change with treatment
Citrulline	− 0.61	< 0.001	↓
Arginine	− 0.47	< 0.001	↓
PC ae C36:4	− 0.46	< 0.001	↓
PC ae C38:5	− 0.42	0.001	↓
PC ae C36:5	− 0.36	0.005	↓
Tyrosine	− 0.34	0.008	↓
PC ae C38:6	− 0.32	0.019	↓
Leucine	0.30	0.040	↑
Proline	0.37	0.011	↑
Isoleucine	0.38	0.005	↑
Alanine	0.42	0.003	↑

^a^Positive beta coefficient indicates a bigger increase in time of the metabolite in the metformin arm compared to placebo arm, whereas negative coefficient indicates a bigger decrease of the metabolite in the metformin arm

^b^*P*-value of the treatment covariate (Metformin vs. Placebo) derived from a multivariate linear model fit on scaled metabolite changes (final evaluation—baseline), adjusted for the scaled baseline value of the metabolite, study center, weight-loss intervention, age, change in BMI, ongoing aromatase-inhibitor therapy, histology, tumor grade, stage, HER2, progesterone receptor, estrogen receptor

### Fatty acids

**Fig. 3:**
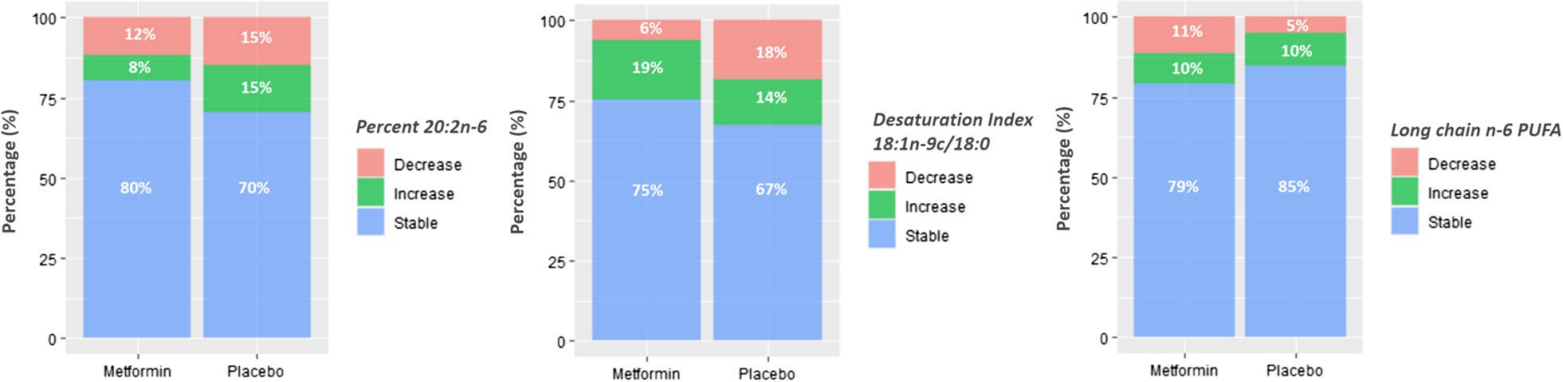
Percent stacked bar-plots of participants with stable, increasing, or decreasing levels of metformin affected fatty acid data over time. Percent stacked bar-plots showing the percentage of participants with stable, increasing or decreasing values over time by treatment group for Percent 20:2n − 6 (left), Desaturation Index 18:1n − 9c/18:0 (center), and Long chain n-6 PUFA (right). Increasing levels of Percent 20:2n − 6 (Metformin vs. Placebo: 8% vs. 15%, *P* = 0.002) and decreasing levels of Desaturation Index 18:1n − 9c/18:0 (Metformin vs. Placebo: 6% vs. 18%, *P* = 0.001) were significantly more frequent in the placebo group. Conversely, Long chain n-6 PUFA decreased significantly more often in the metformin group (Metformin vs. Placebo: 11% vs. 5%, *P* = 0.03)

In multivariate analysis of the fatty acids data, metformin treatment significantly blunted an increase in percent 20:2n − 6 observed in the placebo group (Metformin vs. Placebo: OR = 0.31; 95% CI 0.15–0.65), shown in Fig. 3 a-left. Regarding composite fatty acids, metformin stabilized the Desaturation Index 18:1n − 9c/18:0, which decreased significantly more often in the placebo group (Metformin vs. Placebo: OR = 0.29; 95% CI 0.14–0.61; Fig. 3 a-middle) and decreased long chain n-6 PUFAs (sum of 20:4n − 6, 20:2n − 6, 20:3n − 6, 22:4n − 6, 22:5n − 6) more often than was observed in the placebo group (Metformin vs. Placebo: OR = 1.88; 95% CI 1.07–3.29; Fig. 3 a-right). These results were confirmed in sensitivity analysis, after controlling for baseline BMI instead of BMI change (data not shown).

### Network analysis

**Fig. 4:**
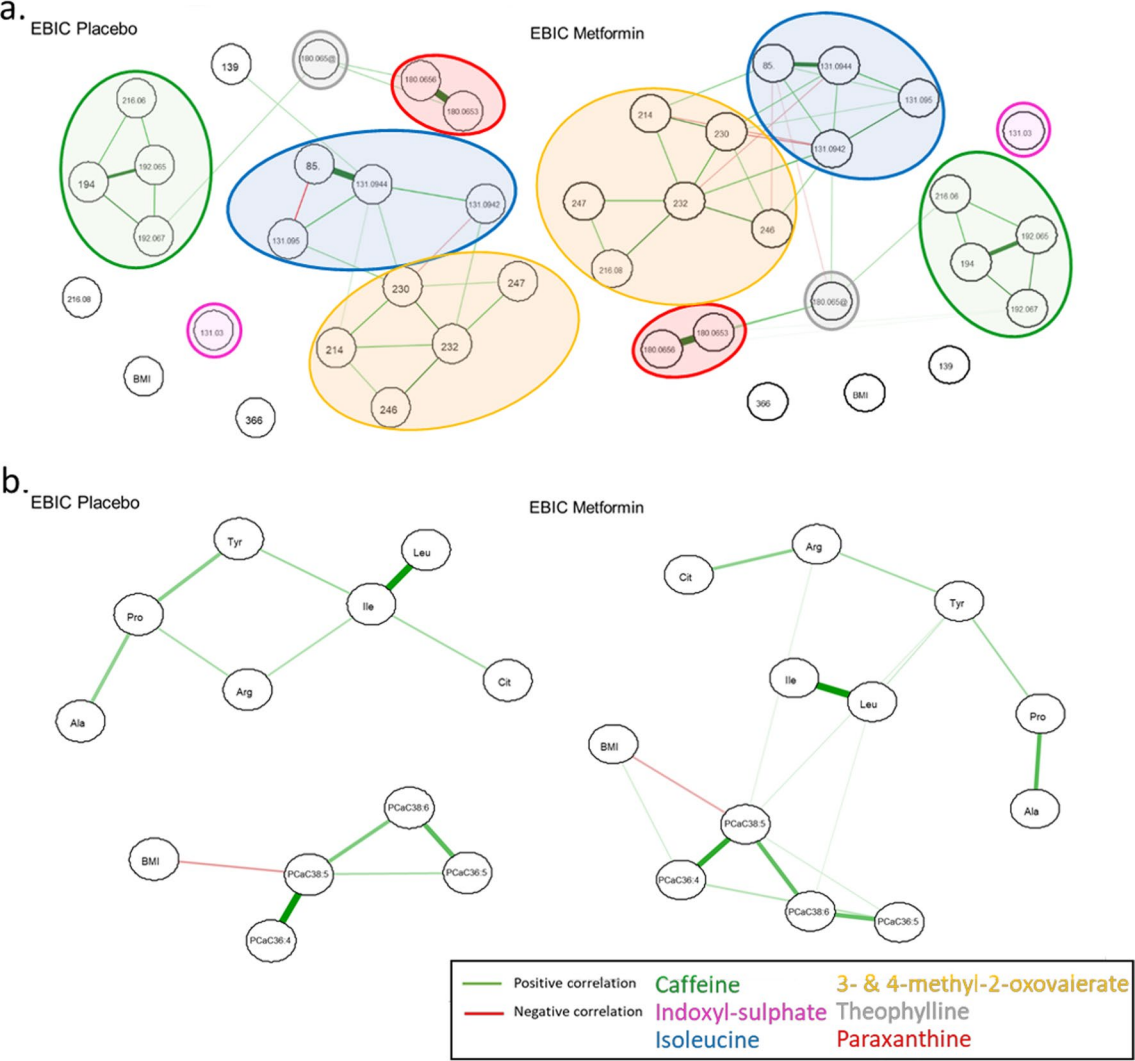
Partial correlation networks of the discriminatory metabolites between treatment groups, applying untargeted and targeted metabolomics. Partial correlation networks including as independent variables the metabolite changes identified as significantly different between treatment groups in multivariate analysis and BMI change, using untargeted (**a**) and targeted metabolomics (**b**). The networks were generated for each treatment group and were estimated based on a graphical LASSO algorithm, with extended Bayesian information criterion (EBIC) to select the model complexity. Nodes are measured variables and edges (lines) are inferred associations (width: strength; color: sign). Green and red edges represent positive and negative correlation, respectively. In networks corresponding to untargeted metabolite changes (**a**), features corresponding to the same metabolite are clustered and encircled in colored spheres with metabolites named in matching color in the legend

KEGG pathway analysis of the 7 annotated metabolites identified in the untargeted analysis pointed to three main pathways, namely caffeine metabolism pathway and valine, leucine and isoleucine biosynthesis and degradation pathways. Leucine (targeted analysis), isoleucine, 3-methyl-2-oxovalerate and 4-methyl-2-oxovalerate (untargeted analysis) are components of the valine, leucine and isoleucine biosynthesis and degradation pathways, with leucine and isoleucine being the precursors of 4-methyl-2-oxovalerate and 3-methyl-2-oxovalerate, respectively in the degradation pathway. Additionally, six of the metabolites highlighted from the targeted analysis, namely arginine, proline, alanine, isoleucine, leucine, and tyrosine are part of the aminoacyl-tRNA biosynthesis pathway. The LASSO algorithm-based partial correlation networks for the metabolites found significant differences between the treatment groups in untargeted and targeted analyses, shown in Fig. 4a, b, respectively.

Caffeine, paraxanthine and theophylline are main components of the caffeine metabolism pathway, with paraxanthine and theophylline the two main metabolites of caffeine formed in the liver. When comparing the ratios of caffeine over its metabolites paraxanthine and theophylline, the ratios were significantly higher after the metformin intervention compared to the placebo condition (Paraxanthine/Caffeine beta = 0.09, *P* = 0.03, Theophylline/Caffeine beta = 0.04, *P* = 0.002, Additional file 4: Fig. S4a), when including the same covariates used in the main analysis. These results were obtained after excluding 19 outliers in Paraxanthine/Caffeine analysis (11 in the metformin group and 8 in the placebo group) and 29 outliers in Theophylline/Caffeine analysis (20 in the metformin group and 9 in the placebo group) that were identified after checking for the residuals of the models for normal distribution. No significant association between the outlier patients excluded from both analyses and the treatment group was observed. Of these outliers, 11/19 from paraxanthine/caffeine analysis and 18/29 from theophylline/caffeine analysis had originally undetected levels of either the numerator or the denominator that were later imputed for the analysis. A detailed overview of the ratio of paraxanthine/caffeine by group and center is provided in Additional file 4: Fig. S4b.

## Discussion

Our metabolomic analyses, using both targeted and untargeted approaches, revealed previously unreported metabolic pathway alterations as well as several previously reported in preclinical models and humans [20, 21]. To the best of our knowledge, this study is the first to assess metabolic responses to metformin treatment via metabolomics in breast cancer survivors. Several classes of metabolites were altered following treatment with metformin, including amino acids such as branched-chain amino acids (BCAAs) and their alpha-ketoacid breakdown products, indoles, xanthines, phosphatidylcholines (PCs) and fatty acids.

Participants treated with metformin had significantly higher levels of the BCAAs leucine and isoleucine and their alpha-keto acid derivatives (BCKAs; 3- and 4-methyl-2-oxovalerate, PubChem CID 47 and 70, respectively), which paradoxically are associated with type 2 diabetes risk and insulin resistance in most studies of metformin naïve subjects [22] but not all [23]. Possible mechanisms underlying our observation include known effects of metformin to decrease the overall activity of mitochondrial BCAA catabolic and oxidative phosphorylation pathways [24], enhance gene expression of sirtuin-1 (SIRT 1), and precipitate AMP-activated protein kinase (AMPK) signaling [23]. Metformin is known to decrease the expression of branched-chain amino acid transaminase (BCAT) 2 [25]. Metformin inhibition of the oxidative phosphorylation pathway complex I results in the accumulation of mitochondrial NADH, which together with elevated NADH generated from fatty acid oxidation in individuals with overweight obesity, negatively feedback on BCAA catabolism at the irreversible, rate-limiting step of BCKA decarboxylation in the mitochondria catalyzed by branched-chain alpha-keto acid dehydrogenase complex (BCKDH) complex. BCKDH has been previously implicated in pro-inflammatory signaling via MAPK [26] as well as in the tumorigenesis of colorectal cancer [27]. Overall effects of metformin to decrease BCAT expression and BCKDH activity may explain the significantly elevated levels of leucine, isoleucine, and their BCKA derivatives. These results merit further investigation of the effects of increased BCAA on tumorigenesis and cancer recurrence.

Effects on metabolism of other amino acid (proline, tyrosine, alanine) were observed in the current study. We observe that proline was significantly increased with metformin treatment, which may be protective against cancer [20, 28]. Interestingly, increased proline dehydrogenase activity has been shown to fuel proline catabolism and consequently lead to increased growth of BC cells in 3D culture and in vivo metastasis formation [29]. We observe that tyrosine was significantly decreased with metformin treatment. Elevated levels of the aromatic amino acid tyrosine are strongly associated with the risk of type 2 diabetes and mitochondrial disfunction [25], while reduced levels of tyrosine are a well-characterized effect of metformin treatment [22]. Higher levels of tyrosine are associated with poor prognosis and therapeutic response [30], and the success of tyrosine kinase inhibitors in cancer treatment and management underscores the clinical relevance of high tyrosine levels [31]. Indeed, a recent randomized phase 2 clinical trial demonstrated that patients with advanced lung adenocarcinoma allocated to a combination tyrosine kinase inhibitor plus metformin treatment demonstrated significantly longer progression free survival as compared to the randomized group receiving only the tyrosine kinase inhibitor [32]. Our finding that tyrosine was significantly lower in participants receiving metformin suggests reduction of tyrosine levels may be one mechanism by which metformin synergistically contributes to tyrosine kinase cancer treatment. We observe that alanine was significantly increased with metformin treatment. Similarly, a recent comparative metabolomics study of circulating prognostic metabolites found a significant inverse association between serum levels of alanine and the risk factor of high mammographic breast cancer density [33], suggesting that elevation of alanine levels during metformin treatment may be a key contributor to its association with cancer risk. Cumulatively, our results commensurate with previously published reports and provide a strong basis for further investigation of metformin’s effects on tyrosine, alanine, and proline and associations with cancer risk reduction.

Previous studies have demonstrated an immediate and sustained decrease in citrulline levels following administration of metformin in humans [34], similar to findings of the current study (FDR-corrected *P* < 0.001). Citrulline is primarily consumed in the kidney as a substrate for arginine synthesis [35], interestingly, arginine levels were also significantly reduced in participants assigned to the metformin arm of the current study (FDR-corrected *P* < 0.001). These changes upon metformin treatment may be explained by diminished citrulline synthesis in the gut [36], lowered hepatic production of citrulline [37], or increased renal uptake of citrulline [38]. Additionally, reduced citrulline levels are associated with increased intestinal permeability [39], which could potentially lead to the increased permeation of gut metabolites, e.g., indoxyl sulfate identified in our study, into the bloodstream.

Significant differences in PC species were also observed in response to metformin treatment. A recent study noted significantly lower levels of the long-chain unsaturated PC ae C36:4 following 4–6 weeks of metformin treatment in individuals with type 2 diabetes (T2D) [34], while an earlier study reported the same finding for PC ae C36:4 in T2D patients under metformin [40]. These findings were paralleled in participants receiving metformin in this study (FDR-corrected *P* < 0.001), thus showing similar metabolic effects of metformin in our population, although less strong. Reports suggest that reductions in PCs and aromatic amino acids (e.g., tyrosine) may not be due to metformin directly, but to the confounding effects of weight loss and subsequently improved metabolic status of study participants [41, 42]. However, this effect would have been minimized in our analyses as BMI was controlled for as a confounding factor. A study investigating the mechanisms of metformin for cancer treatment found that metformin-treated cells exhibited decreased formation of PCs along with a decrease in PC-synthesizing enzymes, and diminished 14C incorporation into fatty acids for membrane synthesis [43]. In our study, levels of four acyl chain PCs were significantly reduced after metformin treatment.

Our fatty acids results suggest metformin treatment effects on several desaturase enzymes, including those encoded by the stearoyl-CoA 9-desaturase genes SCD1 and 5 and the fatty acid desaturase genes FADS5 and 6. Metformin treatment profiles indicate an increase in SCD activity (18:1n9/18:0 desaturation index), however, we are unable to differentiate the enzymatic isoform origin of increased activity (i.e., SCD1 vs. SCD5). SCD activity and association with cancer risk factors in humans varies by cancer type and SCD isoform. Metformin suppresses SCD1 expression via AMPK modulation [44], so the apparent change in stearoyl-CoA 9-desaturase activity that we observe may in fact be due to increased SCD5 activity. A recent study in > 4900 breast cancer patients reports that relatively higher expression of SCD5 improves relapse-free survival [45]. Also, via AMPK activation, metformin treatment reduces the activity index and gene expression of FADS2 [46–48], as observed in our data via greater proportion of metformin-treated participants with reduced percentage of long chain omega-6 fatty acids and stabilized percentage of 20:2n6 compared to that in the placebo group. A more generalized downregulation of desaturase enzymes is observed in metformin-treated cells, with significant reductions both in activity and expression of FADS1-3 [49]. Metformin specifically reduces FADS1 activity [46] and individuals with FADS1 genetic variants with reduced activity have decreased breast cancer risk [50]. Less FADS1 and 2 activity leads to a reduction in long chain omega-6 fatty acids and a less pro-inflammatory tissue milieu that may aid in reducing cancer risk and growth promotion [51]. Future studies should devote more investigational efforts to understanding this intricate relationship between metformin effects on various lipid species, desaturase activities and the consequences for cancer biology.

Metabolite signatures in our analyses indicate an effect of metformin on the gut microbiota. Prior research has shown that metformin influences the activity of gut bacteria and suggests that the metabolic benefit of metformin may in part be mediated by these effects. A recent randomized controlled trial found that germ-free mice which were inoculated with fecal microbiota from humans with type 2 diabetes and receiving metformin treatment, showed significantly improved glucose intolerance [52]. In humans, this association may be explained by the modulatory workings of the estrogen-gut microbiome axis, a bidirectional relationship mediated via the actions of estrogen and β—glucuronidase [53]. Additionally, a recent review noted that specific classes of microbiota-derived metabolites, most notably BCAAs and indole derivatives, have been previously implicated as potential biomarkers in metabolic disorders, such as cancer [54], a finding which was echoed in the current study as well (see significant changes in leucine, isoleucine, tyrosine and indoxyl sulphate in Tables 2, 3). Notably, our results highlight probable perturbations in indole metabolism and aryl hydrocarbon signaling [54], as indicated by significant changes observed in indoxyl sulphate. Future studies would do well to incorporate gut microbiome sequencing to further test this possible association.

An interesting signature of increased caffeine metabolism emerged in the metformin treatment profiles. Caffeine metabolites paraxanthine and theophylline are primarily formed by the enzyme CYP1A2 in the liver. Paraxanthine is the primary metabolite of caffeine (~ 80%) [55] with slower clearance than caffeine. The higher paraxanthine/caffeine and theophylline/caffeine ratios following metformin treatment suggest a higher metabolic activity of CYP1A2. To our knowledge, metformin has not previously been shown to impact the activity of CYP1A2. Metformin is not known to be a substrate of CYP1A2. Although we do not know coffee consumption details for our study participants, potential changes in the habitual consumption of coffee due to the metformin treatment may contribute to the observed changes. We could not find any literature evidence of coffee consumption changes with metformin use. A currently ongoing clinical trial aims to study a six-drug cocktail of probes for CYP enzymes and metformin interactions, including caffeine [56]. The randomized controlled design of our studies minimizes impact of other factors on our observations such as variation in coffee consumption frequency or timing or CYP1A2-modifying drug use across participants.

Nevertheless, the significant association between metformin treatment and decreased levels of caffeine and its downstream metabolites warrants further investigation of CYP1A2 expression and activity given existing evidence supporting a role for CYP1A2 involvement in BC pathogenesis. Notably, CYP1A2 is known to be a key enzyme in BC etiology, contributing variably to carcinogen activation as well as estrogen synthesis and anti-inflammatory pathways [57]. Furthermore, specific isoforms of CYP1A2 have shown reduced activity and led to increased BC risk, whereas the − 3860A variant has consistently demonstrated increased metabolic clearance of caffeine and concomitant reduction in BC risk [57, 58]. Moreover, CYP1A2 activity has also been linked to type 2 diabetes mellitus [59, 60] and has demonstrated interaction effects with coffee consumption and BRCA1 mutation [61, 62]. In addition, CYP1A2 has monoxygenase and epoxygenase activities which result in the generation of anti-inflammatory metabolites of omega-6 and -3 polyunsaturated fatty acids [63–65]. Epoxide metabolites generated from docosapentaenoic acid (DPA) and eicosatetraenoic acid (ETA) have a variety of anti-cancer-related activities in vivo including suppressing inflammation, angiogenesis, and growth and metastasis of human breast and prostate cancer cell lines. Thus, one mechanism potentially underlying the protective effects of metformin may be modulation of CYP1A2.

Strengths of this study include the broad metabolomics approaches utilized, including both targeted and untargeted analyses as well as fatty acid analyses. The study included plasma samples from a large number of participants (n = 373) enrolled in two different randomized controlled trials with sample collections at baseline and study end for 352 participants (paired samples). Limitations of this study include the fact that the two pooled populations come from two different study designs and study centers, which resulted in several baseline differences between the two populations of women, including age, baseline ΒΜΙ and menopausal status. Moreover, the US study also included a weight loss intervention for half the sample, which was not present in the Italian study. These differences lead to the necessity of controlling for several factors in multivariate analysis, reducing the parsimony of the models. However, a series of sensitivity analyses performed in the study and the control for confounders validate the findings and allowed exploration of the effects the BMI, the weight-loss intervention and center adjustments had in the selected models. Furthermore, the pooled analysis including the two different cohorts allowed to generalize the results beyond the single trial, since the results were consistent when they were analyzed by study. Indeed, we observed equidirectional changes in all 11 significant metabolites of the targeted analysis between Italian and USA cohorts, while five of these 11 metabolites retained significance in the Italian cohort. Among the 20 significant features identified by the pooled untargeted analysis, 19 features exhibited changes in the same direction between study cohorts and eight out of 20 features were significant in the smaller Italian cohort. Cumulatively, results between the two study cohorts are well-aligned. Moreover, as in any untargeted metabolomics approach, the need for controlling multiple testing might lead to over-penalization of *P*-values and loss of relevant metabolites [66]. However, the approach of performing targeted analysis in the same set of samples allowed the discovery of additional metabolites, which were not picked up by the untargeted approach. Finally, our methods did not enable identification of the exact composition of the glycerophospholipids detected restricting our ability to interpret the altered phosphatidylcholines’ role in our study.

## Conclusions

In conclusion, our results identify new metabolic effects of metformin treatment that may reduce obesity-related cancer risk. The metformin treatment profiles reflect significant differences in branched chain amino acid catabolism, CYP1A2 activity, phosphatidylcholines and phospholipid metabolism, and lipid desaturase activity that are linked to cancer-promoting pathways. Overall, the metabolomic profiles suggest metformin-associated alterations in mitochondrial activity, liver, kidney, and gut environment (enterocytes, microbiota). This study expands current knowledge regarding potential molecular mechanisms underlying the therapeutic action of metformin in obesity-related metabolic disease and tertiary cancer prevention. Our findings should be validated in further studies on cancer survivors and other populations.

## Supplementary Information

**Additional file 1. Fig. S1**: Scaled scores of the first two components of the Principal Component Analysis of the changes between baseline and final evaluation of the placebo (left) and metformin arm (right), based on the untargeted (upper) and targeted (bottom) metabolomics data.

**Additional file 2. Fig. S2**: Supporting information for the annotations after re-analysing selected study samples and pure chemical standards.

**Additional file 3. Fig. S3**: The trends observed among the six Level 1 annotated metabolites among intervention arm and time point, colored by the study center.

**Additional file 4. Fig. S4**: **a** Boxplots of the paraxanthine/caffeine and theophylline/caffeine ratios, by treatment group and time point (pre- and post-placebo intake, pre- and post-metformin intake). **b** The ratio of Paraxanthine/Caffeine presented by group (Placebo/Metformin) and country (Italy/USA).

**Additional file 5. Fig. S5**: Barplots of the beta regression coefficients of the effect of the treatment (Metformin vs. Placebo) on the scaled metabolite changes identified in the main pooled analysis, estimated in the subgroup analysis including only the Italian sample (in blue) and only the USA sample (in dark yellow), for both targeted metabolomics (**a**) and untargeted metabolomics (**b**).

**Additional file 6. Table S1**: List of quantified metabolites using the AbsoluteIDQ p180 Biocrates kit, organized by chemical classes.

**Additional file 7. Table S2**: List of quantified fatty acids using gas chromatography.

**Additional file 8. Table S3**: Participant baseline characteristics of the Italian and USA cohorts.

**Additional file 9. Table S4**: The 20 features and the annotations achieved, providing the beta regression coefficients of the treatment covariate effect on feature changes in time and the corrected *P*-values.

## Data Availability

The data underlying this article may be shared upon reasonable request to the corresponding author, following approval from the involved Research Institutions.

## References

[1] Sung H, Ferlay J, Siegel RL, Laversanne M, Soerjomataram I, Jemal A, Bray F, Global Cancer Statistics (2020). GLOBOCAN estimates of incidence and mortality worldwide for 36 cancers in 185 countries. CA Cancer J Clin.

[2] Miller KD, Nogueira L, Mariotto AB, Rowland JH, Yabroff KR, Alfano CM, Jemal A, Kramer JL, Siegel RL (2019). Cancer treatment and survivorship statistics, 2019. CA Cancer J Clin.

[3] Siegel RL, Miller KD, Fuchs HE, Jemal A (2021). Cancer statistics, 2021. CA Cancer J Clin.

[4] Picon-Ruiz M, Morata-Tarifa C, Valle-Goffin JJ, Friedman ER, Slingerland JM (2017). Obesity and adverse breast cancer risk and outcome: mechanistic insights and strategies for intervention. CA Cancer J Clin.

[5] Yerevanian A, Soukas AA (2019). Metformin: mechanisms in human obesity and weight loss. Curr Obes Rep.

[6] Pernicova I, Korbonits M (2014). Metformin–mode of action and clinical implications for diabetes and cancer. Nat Rev Endocrinol.

[7] Madiraju AK, Qiu Y, Perry RJ, Rahimi Y, Zhang X-M, Zhang D, Camporez J-PG, Cline GW, Butrico GM, Kemp BE, Casals G, Steinberg GR, Vatner DF, Petersen KF, Shulman GI (2018). Metformin inhibits gluconeogenesis via a redox-dependent mechanism in vivo. Nat Med.

[8] Agius L, Ford BE, Chachra SS (2020). The metformin mechanism on gluconeogenesis and AMPK activation: the metabolite perspective. Int J Mol Sci.

[9] Madiraju AK, Erion DM, Rahimi Y, Zhang X-M, Braddock DT, Albright RA, Prigaro BJ, Wood JL, Bhanot S, MacDonald MJ, Jurczak MJ, Camporez J-P, Lee H-Y, Cline GW, Samuel VT, Kibbey RG, Shulman GI (2014). Metformin suppresses gluconeogenesis by inhibiting mitochondrial glycerophosphate dehydrogenase. Nature.

[10] Sun L, Xie C, Wang G, Wu Y, Wu Q, Wang X, Liu J, Deng Y, Xia J, Chen B, Zhang S, Yun C, Lian G, Zhang X, Zhang H, Bisson WH, Shi J, Gao X, Ge P, Liu C, Krausz KW, Nichols RG, Cai J, Rimal B, Patterson AD, Wang X, Gonzalez FJ, Jiang C (2018). Gut microbiota and intestinal FXR mediate the clinical benefits of metformin. Nat Med.

[11] Patterson RE, Marinac CR, Sears DD, Kerr J, Hartman SJ, Cadmus-Bertram L, Villaseñor A, Flatt SW, Godbole S, Li H, Laughlin GA, Oratowski-Coleman J, Parker BA, Natarajan L (2018). The effects of metformin and weight loss on biomarkers associated with breast cancer outcomes. JNCI J Natl Cancer Inst.

[12] Patterson RE, Marinac CR, Natarajan L, Hartman SJ, Cadmus-Bertram L, Flatt SW, Li H, Parker B, Oratowski-Coleman J, Villaseñor A, Godbole S, Kerr J (2016). Recruitment strategies, design, and participant characteristics in a trial of weight-loss and metformin in breast cancer survivors. Contemp Clin Trials.

[13] Geijsen AJMR, Brezina S, Keski-Rahkonen P, Baierl A, Bachleitner-Hofmann T, Bergmann MM, Boehm J, Brenner H, Chang-Claude J, van Duijnhoven FJB, Gigic B, Gumpenberger T, Hofer P, Hoffmeister M, Holowatyj AN, Karner-Hanusch J, Kok DE, Leeb G, Ulvik A, Robinot N, Ose J, Stift A, Schrotz-King P, Ulrich AB, Ueland PM, Kampman E, Scalbert A, Habermann N, Gsur A, Ulrich CM (2019). Plasma metabolites associated with colorectal cancer: a discovery-replication strategy. Int J Cancer.

[14] Friedman J, Hastie T, Tibshirani R (2008). Sparse inverse covariance estimation with the graphical lasso. Biostatistics.

[15] Wishart DS, Feunang YD, Marcu A, Guo AC, Liang K, Vázquez-Fresno R, Sajed T, Johnson D, Li C, Karu N, Sayeeda Z, Lo E, Assempour N, Berjanskii M, Singhal S, Arndt D, Liang Y, Badran H, Grant J, Serra-Cayuela A, Liu Y, Mandal R, Neveu V, Pon A, Knox C, Wilson M, Manach C, Scalbert A (2018). HMDB 4.0: the human metabolome database for 2018. Nucl Acids Res.

[16] Li L, Li R, Zhou J, Zuniga A, Stanislaus AE, Wu Y, Huan T, Zheng J, Shi Y, Wishart DS, Lin G (2013). MyCompoundID: using an evidence-based metabolome library for metabolite identification. Anal Chem.

[17] Sumner LW, Amberg A, Barrett D, Beale MH, Beger R, Daykin CA, Fan TW-M, Fiehn O, Goodacre R, Griffin JL, Hankemeier T, Hardy N, Harnly J, Higashi R, Kopka J, Lane AN, Lindon JC, Marriott P, Nicholls AW, Reily MD, Thaden JJ, Viant MR (2007). Proposed minimum reporting standards for chemical analysis Chemical Analysis Working Group (CAWG) Metabolomics Standards Initiative (MSI). Metabol Off J Metabolomic Soc.

[18] Chajès V, Assi N, Biessy C, Ferrari P, Rinaldi S, Slimani N, Lenoir GM, Baglietto L, His M, Boutron-Ruault MC, Trichopoulou A, Lagiou P, Katsoulis M, Kaaks R, Kühn T, Panico S, Pala V, Masala G, Bueno-de-Mesquita HB, Peeters PH, van Gils C, Hjartåker A, Standahl Olsen K, Borgund Barnung R, Barricarte A, Redondo-Sanchez D, Menéndez V, Amiano P, Wennberg M, Key T, Khaw KT, Merritt MA, Riboli E, Gunter MJ, Romieu I (2017). A prospective evaluation of plasma phospholipid fatty acids and breast cancer risk in the EPIC study. Ann. Oncol. Off. J. Eur. Soc. Med. Oncol..

[19] Tibshirani R (1996). Regression shrinkage and selection via the lasso. J R Stat Soc Ser B Methodol.

[20] Wei Y, Jasbi P, Shi X, Turner C, Hrovat J, Liu L, Rabena Y, Porter P, Gu H (2021). Early breast cancer detection using untargeted and targeted metabolomics. J Proteome Res.

[21] Zhang L, Han J (2017). Branched-chain amino acid transaminase 1 (BCAT1) promotes the growth of breast cancer cells through improving mTOR-mediated mitochondrial biogenesis and function. Biochem Biophys Res Commun.

[22] Safai N, Suvitaival T, Ali A, Spégel P, Al-Majdoub M, Carstensen B, Vestergaard H, Ridderstråle M (2018). CIMT Trial Group, effect of metformin on plasma metabolite profile in the Copenhagen Insulin and Metformin Therapy (CIMT) trial. Diabet Med J Br Diabet Assoc.

[23] Ye Z, Wang S, Zhang C, Zhao Y (2020). Coordinated modulation of energy metabolism and inflammation by branched-chain amino acids and fatty acids. Front Endocrinol.

[24] Martínez-Reyes I, Chandel NS (2020). Mitochondrial TCA cycle metabolites control physiology and disease. Nat Commun.

[25] Sonnet DS, O’Leary MN, Gutierrez MA, Nguyen SM, Mateen S, Hsu Y, Mitchell KP, Lopez AJ, Vockley J, Kennedy BK, Ramanathan A (2016). Metformin inhibits Branched Chain Amino Acid (BCAA) derived ketoacidosis and promotes metabolic homeostasis in MSUD. Sci Rep.

[26] Peng H, Wang Y, Luo W (2020). Multifaceted role of branched-chain amino acid metabolism in cancer. Oncogene.

[27] Xue P, Zeng F, Duan Q, Xiao J, Liu L, Yuan P, Fan L, Sun H, Malyarenko OS, Lu H, Xiu R, Liu S, Shao C, Zhang J, Yan W, Wang Z, Zheng J, Zhu F (2017). BCKDK of BCAA catabolism cross-talking with the MAPK pathway promotes tumorigenesis of colorectal cancer. EBioMedicine.

[28] Jasbi P, Wang D, Cheng SL, Fei Q, Cui JY, Liu L, Wei Y, Raftery D, Gu H (2019). Breast cancer detection using targeted plasma metabolomics. J Chromatogr B Analyt Technol Biomed Life Sci.

[29] Elia I, Broekaert D, Christen S, Boon R, Radaelli E, Orth MF, Verfaillie C, Grünewald TGP, Fendt S-M (2017). Proline metabolism supports metastasis formation and could be inhibited to selectively target metastasizing cancer cells. Nat Commun.

[30] Ha JR, Siegel PM, Ursini-Siegel J (2016). The tyrosine kinome dictates breast cancer heterogeneity and therapeutic responsiveness. J Cell Biochem.

[31] Takahashi H, Isogawa M (2018). Management of breast cancer brain metastases. Chin Clin Oncol.

[32] Arrieta O, Barrón F, Padilla M-ÁS, Avilés-Salas A, Ramírez-Tirado LA, Arguelles Jiménez MJ, Vergara E, Zatarain-Barrón ZL, Hernández-Pedro N, Cardona AF, Cruz-Rico G, Barrios-Bernal P, Yamamoto Ramos M, Rosell R (2019). Effect of metformin plus tyrosine kinase inhibitors compared with tyrosine kinase inhibitors alone in patients with epidermal growth factor receptor-mutated lung adenocarcinoma: a phase 2 randomized clinical trial. JAMA Oncol.

[33] Bendinelli B, Vignoli A, Palli D, Assedi M, Ambrogetti D, Luchinat C, Caini S, Saieva C, Turano P, Masala G (2021). Prediagnostic circulating metabolites in female breast cancer cases with low and high mammographic breast density. Sci Rep.

[34] Breier M, Wahl S, Prehn C, Ferrari U, Sacco V, Weise M, Grallert H, Adamski J, Lechner A (2017). Immediate reduction of serum citrulline but no change of steroid profile after initiation of metformin in individuals with type 2 diabetes. J Steroid Biochem Mol Biol.

[35] van de Poll MCG, Soeters PB, Deutz NEP, Fearon KCH, Dejong CHC (2004). Renal metabolism of amino acids: its role in interorgan amino acid exchange. Am J Clin Nutr.

[36] Li LO, Hu Y-F, Wang L, Mitchell M, Berger A, Coleman RA (2010). Early hepatic insulin resistance in mice: a metabolomics analysis. Mol Endocrinol Baltim Md.

[37] Davis BJ, Xie Z, Viollet B, Zou M-H (2006). Activation of the AMP-activated kinase by antidiabetes drug metformin stimulates nitric oxide synthesis in vivo by promoting the association of heat shock protein 90 and endothelial nitric oxide synthase. Diabetes.

[38] Irving BA, Carter RE, Soop M, Weymiller A, Syed H, Karakelides H, Bhagra S, Short KR, Tatpati L, Barazzoni R, Nair KS (2015). Effect of insulin sensitizer therapy on amino acids and their metabolites. Metabolism.

[39] Batista MA, Nicoli JR, dos SantosMartins F, Nogueira Machado JA, Esteves Arantes RM, Pacífico Quirino IE, Davisson Correia MIT, Cardoso VN (2012). Pretreatment with citrulline improves gut barrier after intestinal obstruction in mice. J Parenter Enter Nutr.

[40] Xu T, Brandmaier S, Messias AC, Herder C, Draisma HHM, Demirkan A, Yu Z, Ried JS, Haller T, Heier M, Campillos M, Fobo G, Stark R, Holzapfel C, Adam J, Chi S, Rotter M, Panni T, Quante AS, He Y, Prehn C, Roemisch-Margl W, Kastenmüller G, Willemsen G, Pool R, Kasa K, van Dijk KW, Hankemeier T, Meisinger C, Thorand B, Ruepp A, Hrabé de Angelis M, Li Y, Wichmann H-E, Stratmann B, Strauch K, Metspalu A, Gieger C, Suhre K, Adamski J, Illig T, Rathmann W, Roden M, Peters A, van Duijn CM, Boomsma DI, Meitinger T, Wang-Sattler R (2015). Effects of metformin on metabolite profiles and LDL cholesterol in patients with type 2 diabetes. Diabetes Care.

[41] Floegel A, Stefan N, Yu Z, Mühlenbruch K, Drogan D, Joost H-G, Fritsche A, Häring H-U, Hrabě de Angelis M, Peters A, Roden M, Prehn C, Wang-Sattler R, Illig T, Schulze MB, Adamski J, Boeing H, Pischon T (2013). Identification of serum metabolites associated with risk of type 2 diabetes using a targeted metabolomic approach. Diabetes.

[42] Kwee LC, Ilkayeva O, Muehlbauer MJ, Bihlmeyer N, Wolfe B, Purnell JQ, Xavier Pi-Sunyer F, Chen H, Bahnson J, Newgard CB, Shah SH, Laferrère B (2021). Metabolites and diabetes remission after weight loss. Nutr Diabetes.

[43] Smith TAD, Phyu SM (2016). Metformin decouples phospholipid metabolism in breast cancer cells. PLoS ONE.

[44] Kim E, Liu N-C, Yu I-C, Lin H-Y, Lee Y-F, Sparks JD, Chen L-M, Chang C (2011). Metformin inhibits nuclear receptor TR4-mediated hepatic stearoyl-CoA desaturase 1 gene expression with altered insulin sensitivity. Diabetes.

[45] Zhao W, Sun L, Li X, Wang J, Zhu Y, Jia Y, Tong Z (2021). SCD5 expression correlates with prognosis and response to neoadjuvant chemotherapy in breast cancer. Sci Rep.

[46] Miklankova D, Markova I, Hüttl M, Zapletalova I, Poruba M, Malinska H (2021). Metformin affects cardiac arachidonic acid metabolism and cardiac lipid metabolite storage in a prediabetic rat model. Int J Mol Sci.

[47] Gandini S, Puntoni M, Heckman-Stoddard BM, Dunn BK, Ford L, DeCensi A, Szabo E (2014). Metformin and cancer risk and mortality: a systematic review and meta-analysis taking into account biases and confounders. Cancer Prev Res Phila Pa.

[48] Azrad M, Zhang K, Vollmer RT, Madden J, Polascik TJ, Snyder DC, Ruffin MT, Moul JW, Brenner D, Hardy RW, Demark-Wahnefried W (2012). Prostatic alpha-linolenic acid (ALA) is positively associated with aggressive prostate cancer: a relationship which may depend on genetic variation in ALA metabolism. PLoS ONE.

[49] Kim W, Deik A, Gonzalez C, Gonzalez ME, Fu F, Ferrari M, Churchhouse CL, Florez JC, Jacobs SBR, Clish CB, Rhee EP (2019). Polyunsaturated fatty acid desaturation is a mechanism for glycolytic NAD+ recycling. Cell Metab.

[50] Preethika A, Sonkusare S, Suchetha Kumari N (2022). Single nucleotide polymorphism of fatty acid desaturase gene and breast cancer risk in estrogen receptor subtype. Gene.

[51] McCarty MF, DiNicolantonio JJ (2018). Minimizing membrane arachidonic acid content as a strategy for controlling cancer: a review. Nutr Cancer.

[52] Wu H, Esteve E, Tremaroli V, Khan MT, Caesar R, Mannerås-Holm L, Ståhlman M, Olsson LM, Serino M, Planas-Fèlix M, Xifra G, Mercader JM, Torrents D, Burcelin R, Ricart W, Perkins R, Fernàndez-Real JM, Bäckhed F (2017). Metformin alters the gut microbiome of individuals with treatment-naive type 2 diabetes, contributing to the therapeutic effects of the drug. Nat Med.

[53] Baker JM, Al-Nakkash L, Herbst-Kralovetz MM (2017). Estrogen-gut microbiome axis: physiological and clinical implications. Maturitas.

[54] Agus A, Clément K, Sokol H (2021). Gut microbiota-derived metabolites as central regulators in metabolic disorders. Gut.

[55] Carrillo JA, Christensen M, Ramos SI, Alm C, Dahl ML, Benitez J, Bertilsson L (2000). Evaluation of caffeine as an in vivo probe for CYP1A2 using measurements in plasma, saliva, and urine. Ther Drug Monit.

[56] Metformin’s Effect on Drug Metabolism in Patients With Type 2 Diabetes—Full Text View—ClinicalTrials.gov, (n.d.). https://clinicaltrials.gov/ct2/show/NCT04504045 (accessed May 12, 2022).

[57] Ayari I, Fedeli U, Saguem S, Hidar S, Khlifi S, Pavanello S (2013). Role of CYP1A2 polymorphisms in breast cancer risk in women. Mol Med Rep.

[58] Imene A, Maurice AJ, Arij M, Sofia P, Saad S (2015). Breast cancer association with CYP1A2 activity and gene polymorphisms—a preliminary case-control study in Tunisia. Asian Pac J Cancer Prev APJCP.

[59] Elfaki I, Mir R, Almutairi FM, Duhier FMA (2018). Cytochrome P450: polymorphisms and roles in cancer, diabetes and atherosclerosis. Asian Pac J Cancer Prev.

[60] Matzke GR, Frye RF, Early JJ, Straka RJ, Carson SW (2000). Evaluation of the influence of diabetes mellitus on antipyrine metabolism and CYP1A2 and CYP2D6 activity. Pharmacotherapy.

[61] Urry E, Jetter A, Landolt H-P (2016). Assessment of CYP1A2 enzyme activity in relation to type-2 diabetes and habitual caffeine intake. Nutr Metab.

[62] Kotsopoulos J, Ghadirian P, El-Sohemy A, Lynch HT, Snyder C, Daly M, Domchek S, Randall S, Karlan B, Zhang P, Zhang S, Sun P, Narod SA (2007). The CYP1A2 genotype modifies the association between coffee consumption and breast cancer risk among BRCA1 mutation carriers. Cancer Epidemiol Biomark Prev Publ Am Assoc Cancer Res Cosponsored Am Soc Prev Oncol.

[63] Westphal C, Konkel A, Schunck W-H (2011). CYP-eicosanoids—a new link between omega-3 fatty acids and cardiac disease?. Prostaglandins Other Lipid Mediat.

[64] Zhang G, Kodani S, Hammock BD (2014). Stabilized epoxygenated fatty acids regulate inflammation, pain, angiogenesis and cancer. Prog Lipid Res.

[65] He J, Wang C, Zhu Y, Ai D (2016). Soluble epoxide hydrolase: a potential target for metabolic diseases. J Diabetes.

[66] Vinaixa M, Samino S, Saez I, Duran J, Guinovart JJ, Yanes O (2012). A guideline to univariate statistical analysis for LC/MS-based untargeted metabolomics-derived data. Metabolites.

