# Supplementary material for: Metabolomic profiles of metformin in breast cancer survivors: a pooled analysis of plasmas from two randomized placebo-controlled trials
Source: J Transl Med. 2022 Dec 29;20:629. doi: 10.1186/s12967-022-03809-6 (PMC9798585; doi:10.1186/s12967-022-03809-6)
Supplement: Supplementary file 1 — Additional file 1. Fig. S1: Scaled scores of the first two components of the Principal Component Analysis of the changes between baseline and final evaluation of the placebo (left) and metformin arm (right), based on the untargeted (upper) and targeted (bottom) metabolomics data. [file 12967_2022_3809_MOESM1_ESM.docx]

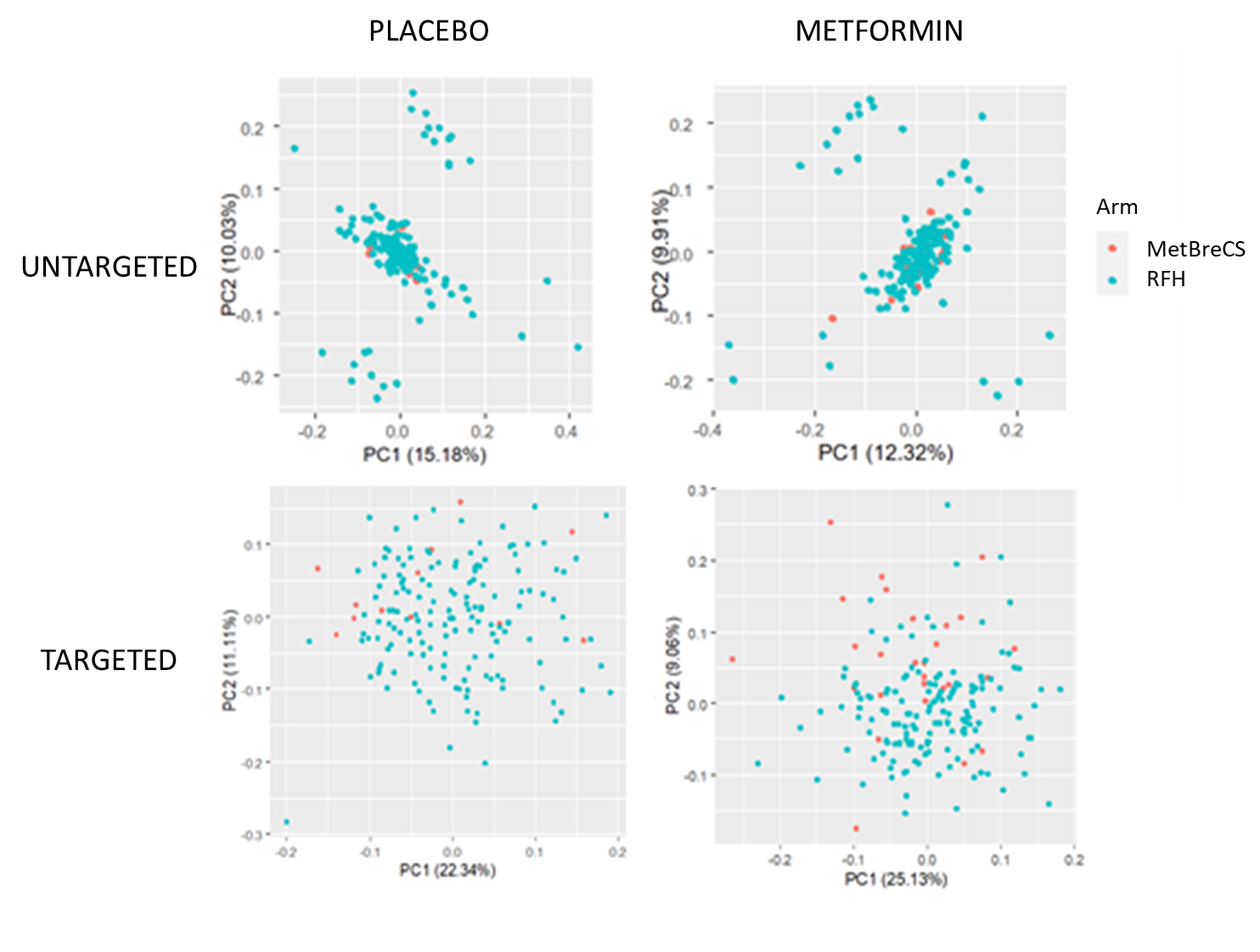


**Supplementary Figure S1.** Scaled scores of the first two components of the Principal Component Analysis of the changes between baseline and final evaluation of the placebo (left) and metformin arm (right), based on the untargeted (upper) and targeted (bottom) metabolomics data.
