# Supplementary material for: Metabolomic profiles of metformin in breast cancer survivors: a pooled analysis of plasmas from two randomized placebo-controlled trials
Source: J Transl Med. 2022 Dec 29;20:629. doi: 10.1186/s12967-022-03809-6 (PMC9798585; doi:10.1186/s12967-022-03809-6)

**
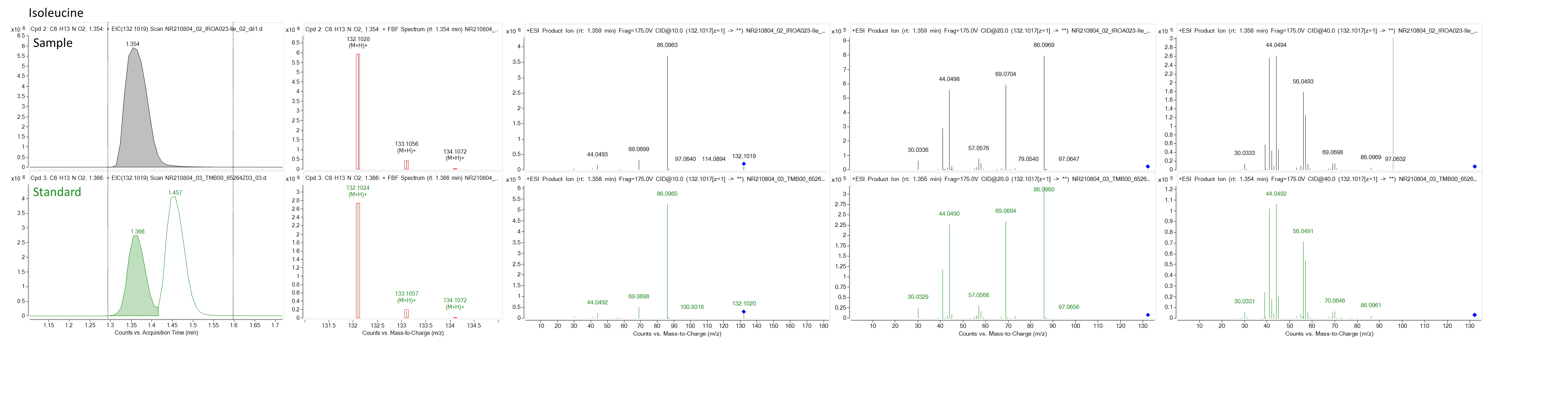

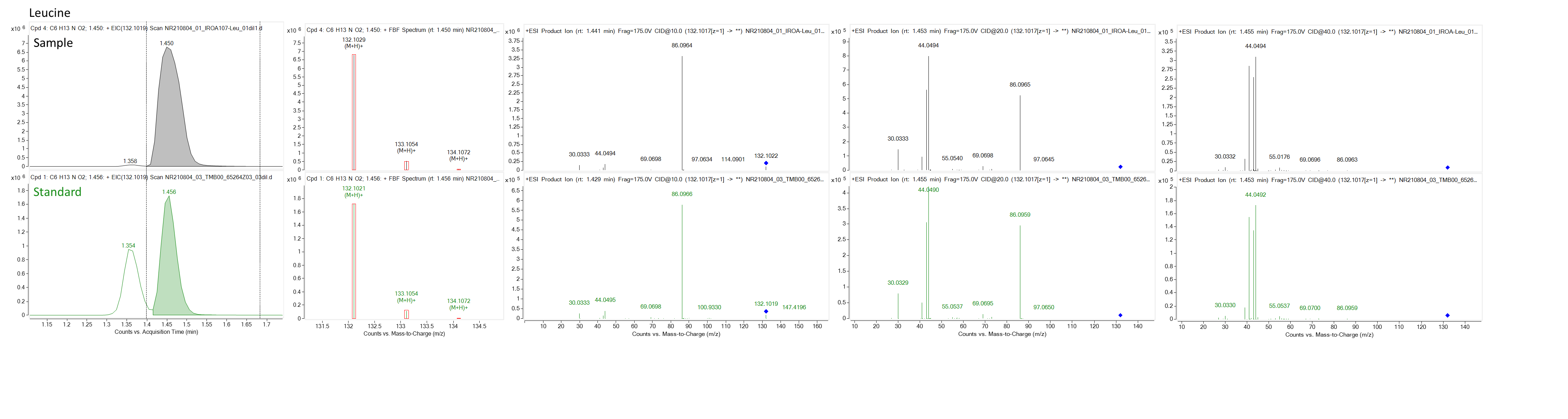
Supplementary Figure S2** Supporting information for the annotations after re-analysing selected study samples and pure chemical standards. From left to right for each metabolite are provided: the corresponding chromatograms of the selected chemical formula of the metabolite of interest, the isotopic patterns (the red rectangles depict the expected isotopic pattern of the corresponding elemental composition, while the black lines are the observed isotopic pattern), and the fragmentation patterns at 10, 20 and 40 V.


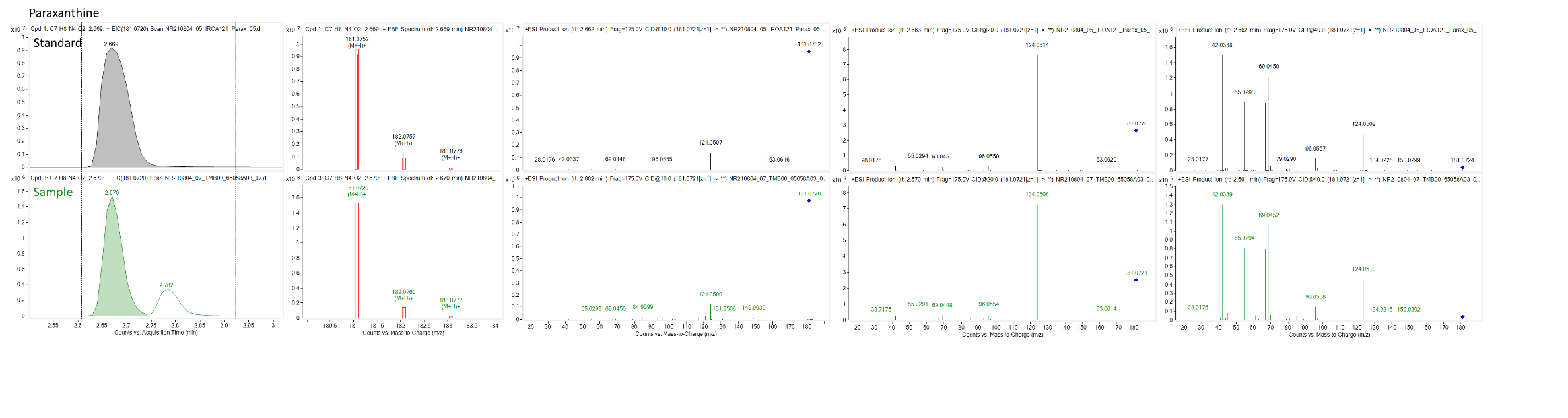

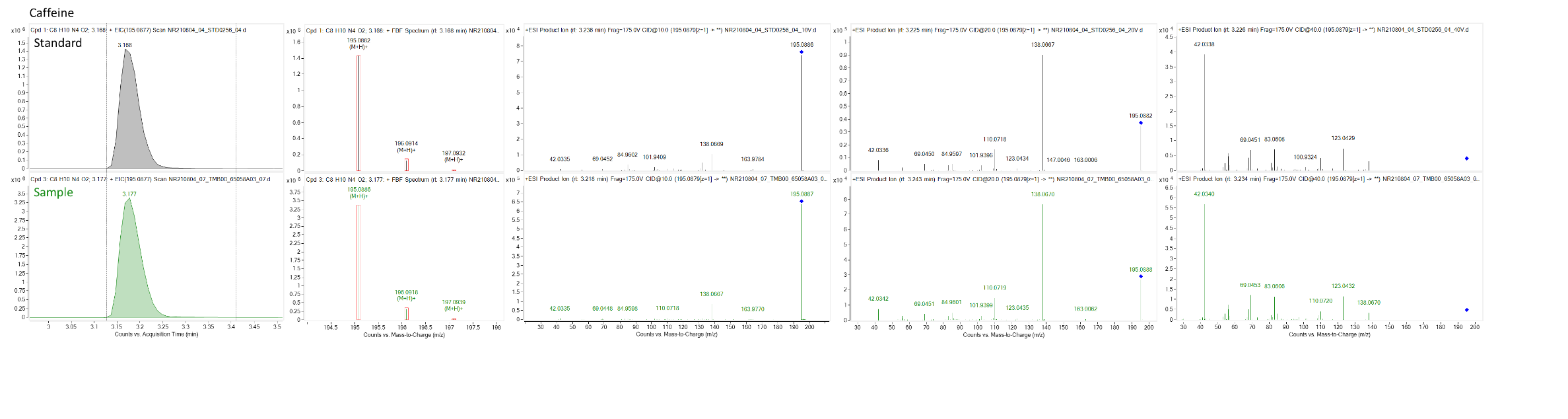

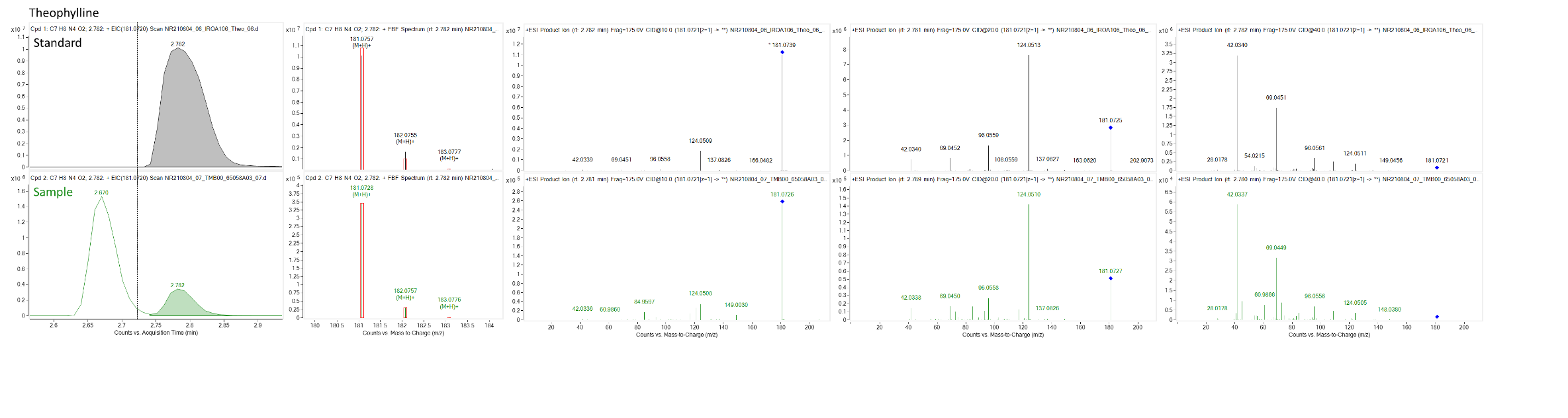


**Supplementary Figure S2** (continue)


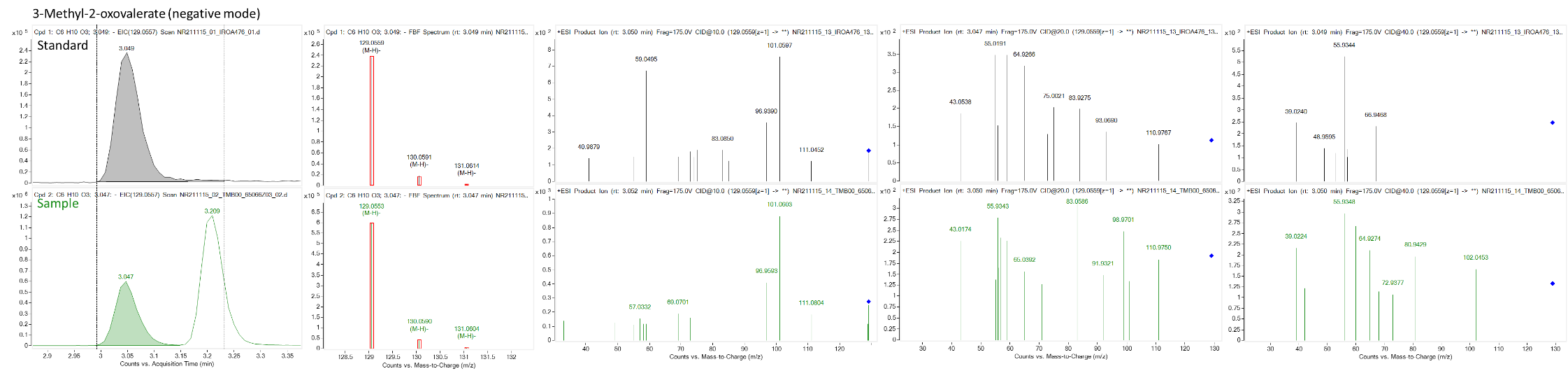

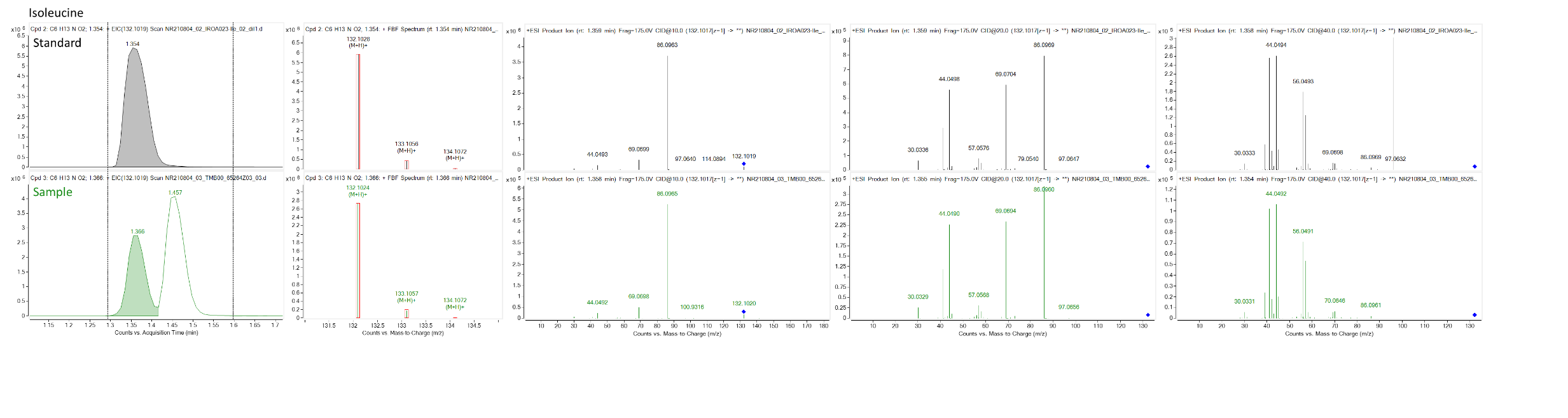


**Supplementary Figure S2** (continue)


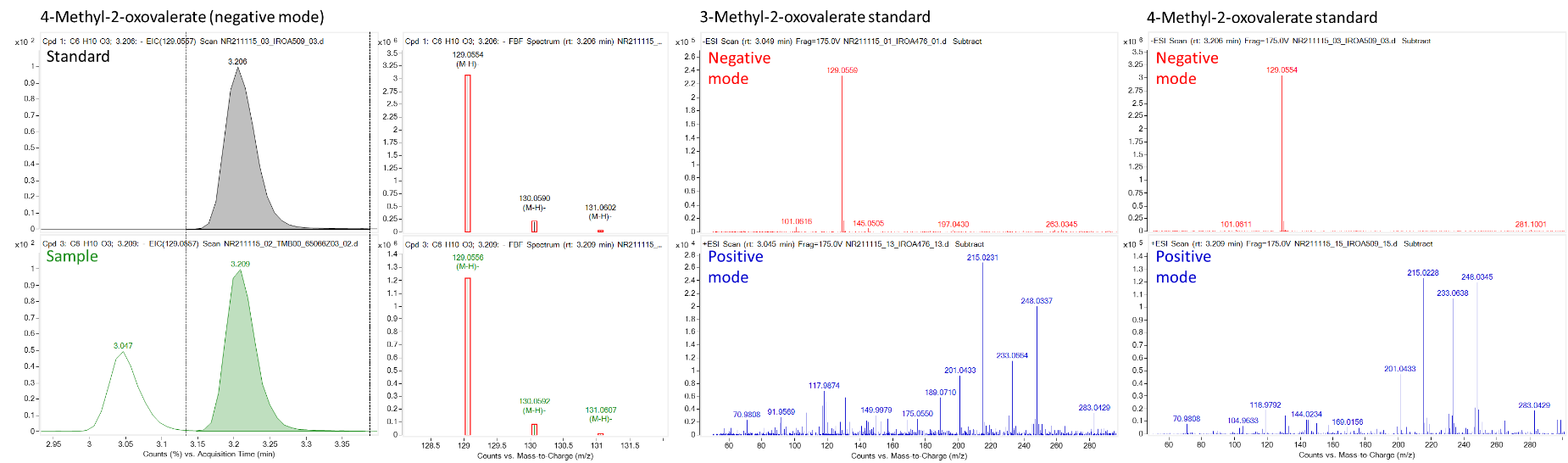
* The identification of 6 features as two isomer metabolites, namely 3-methyl-2-oxovalerate and 4-methyl-2-oxovalerate, as well as of the feature identified as indoxyl sulfate was classified as Level 1, however, it was achieved only after re-analysis of selected samples and the corresponding standards on negative mode. The three corresponding analytical standards were analyzed also on positive mode, but no peak was detected matching their chemical formula.


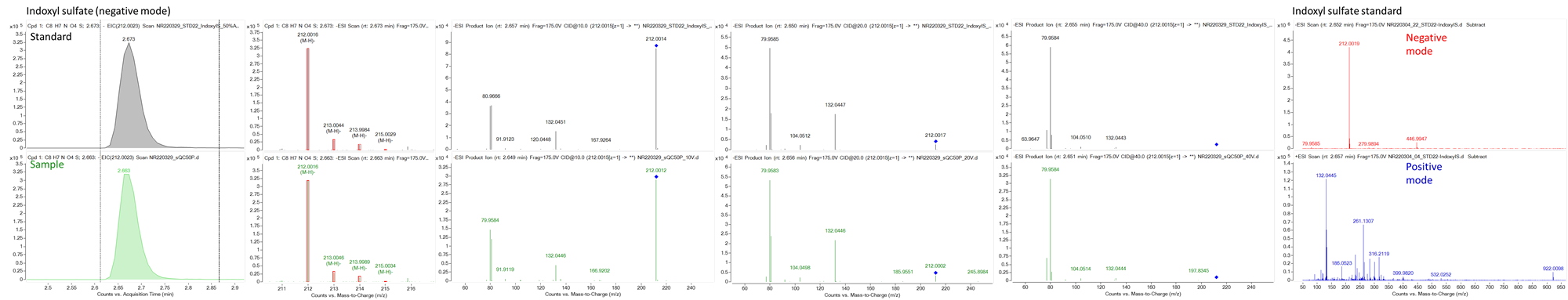


It is worth noting that the re-analysis of standards was performed months after the initial analysis of the samples and some retention time shifts are observed. For clarification, the corresponding chromatograms of initial analysis and identification-related re-analysis in positive mode are provided below:


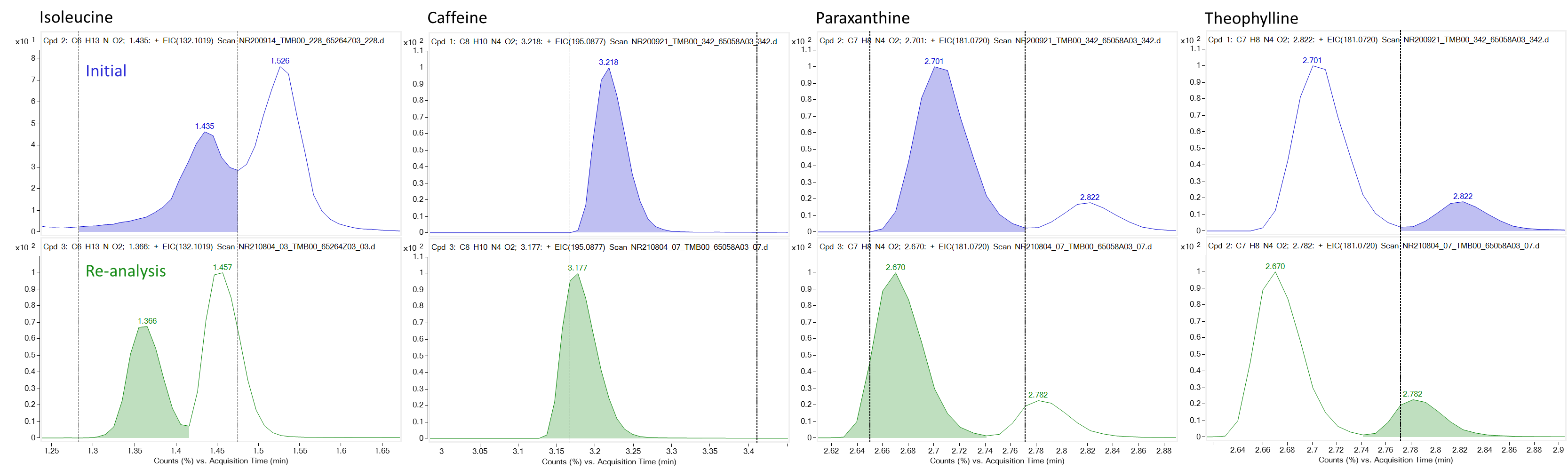

Supplement: Supplementary file 2 — Additional file 2. Fig. S2: Supporting information for the annotations after re-analysing selected study samples and pure chemical standards. [file 12967_2022_3809_MOESM2_ESM.docx]
