# Supplementary material for: Metabolomic profiles of metformin in breast cancer survivors: a pooled analysis of plasmas from two randomized placebo-controlled trials
Source: J Transl Med. 2022 Dec 29;20:629. doi: 10.1186/s12967-022-03809-6 (PMC9798585; doi:10.1186/s12967-022-03809-6)
Supplement: Supplementary file 3 — Additional file 3. Fig. S3: The trends observed among the six Level 1 annotated metabolites among intervention arm and time point, colored by the study center. [file 12967_2022_3809_MOESM3_ESM.docx]

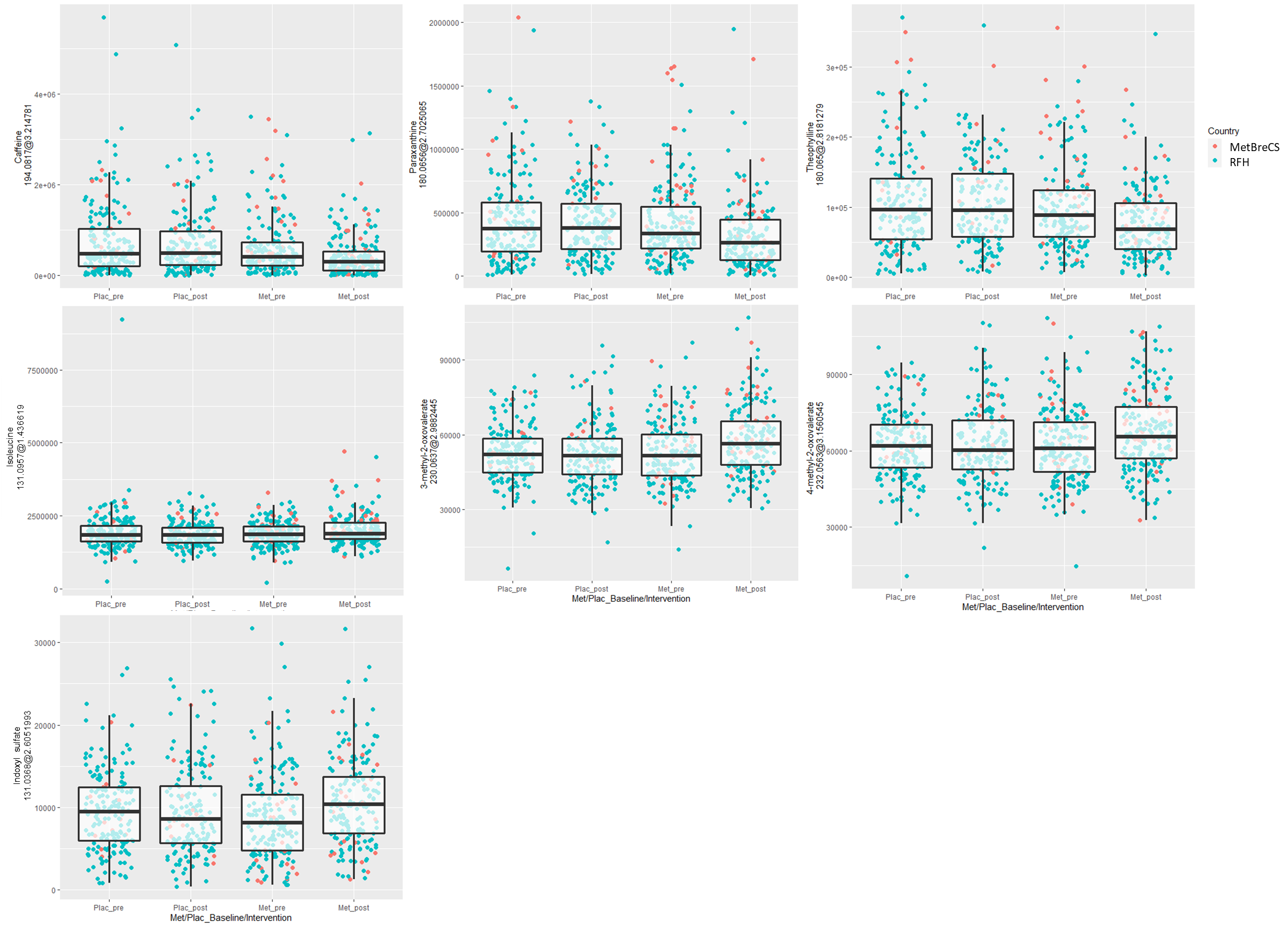


**Supplementary Figure S3** Trends observed among the six Level 1 annotated metabolites among intervention arm and time point, colored by the study center.
