# Supplementary material for: Metabolomic profiles of metformin in breast cancer survivors: a pooled analysis of plasmas from two randomized placebo-controlled trials
Source: J Transl Med. 2022 Dec 29;20:629. doi: 10.1186/s12967-022-03809-6 (PMC9798585; doi:10.1186/s12967-022-03809-6)
Supplement: Supplementary file 4 — Additional file 4. Fig. S4: a Boxplots of the paraxanthine/caffeine and theophylline/caffeine ratios, by treatment group and time point (pre- and post-placebo intake, pre- and post-metformin intake). b The ratio of Paraxanthine/Caffeine presented by group (Placebo/Metformin) and country (Italy/USA). [file 12967_2022_3809_MOESM4_ESM.docx]

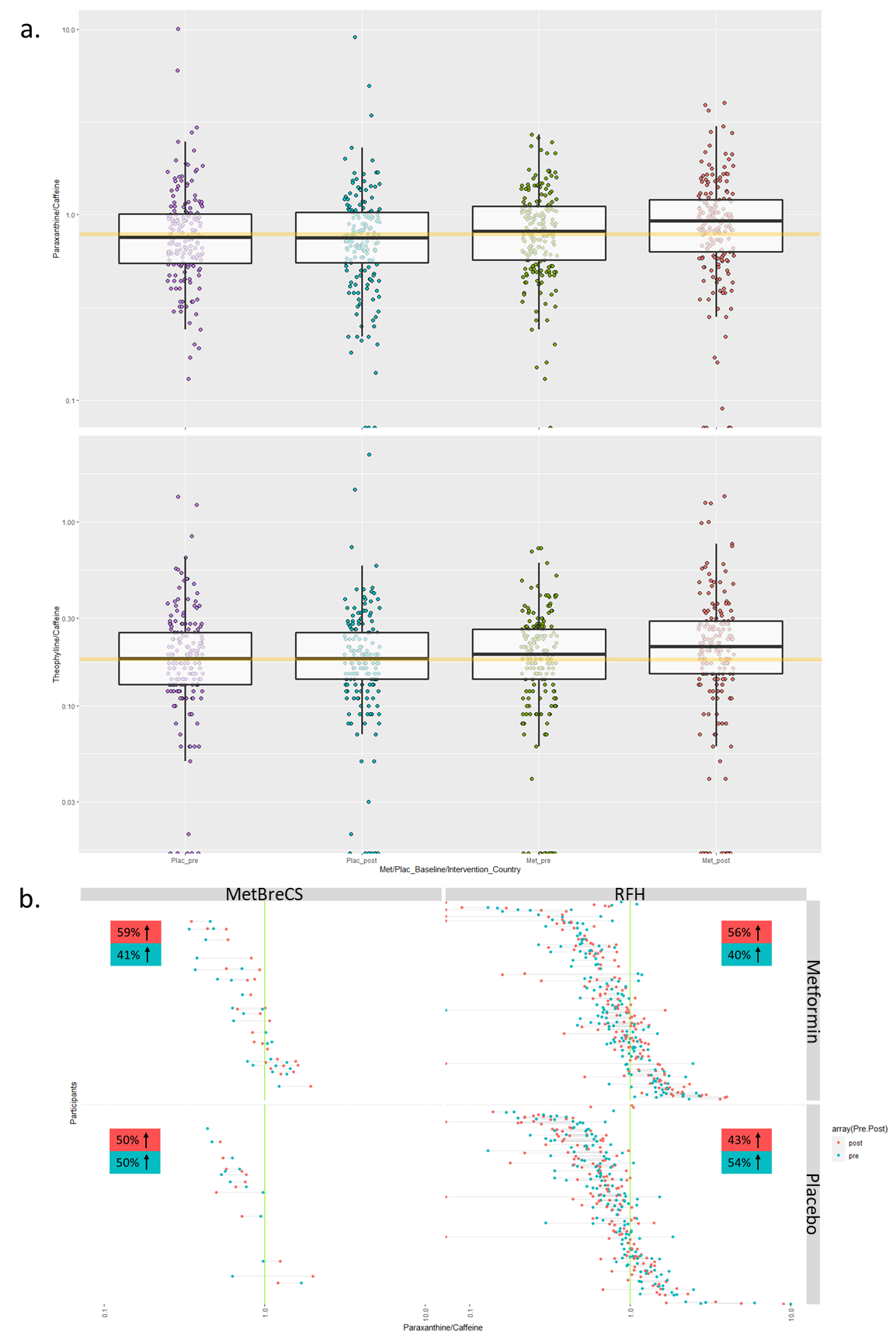


**Supplementary Figure S4**

**a.** Boxplots of the paraxanthine/caffeine and theophylline/caffeine ratios, by treatment group and time point (pre- and post- placebo intake, pre- and post- metformin intake).

**b.** The ratio of Paraxanthine/Caffeine presented by group (Placebo/Metformin) and country (Italy/USA). In the graph, each connected group of dots represents a participant, the color of the dot corresponds to the different time points and the percentages represent the number of sets that the ratio of paraxanthine/caffeine was higher in the pre- (blue) or post- (red) time point.
