# Supplementary material for: Metabolomic profiles of metformin in breast cancer survivors: a pooled analysis of plasmas from two randomized placebo-controlled trials
Source: J Transl Med. 2022 Dec 29;20:629. doi: 10.1186/s12967-022-03809-6 (PMC9798585; doi:10.1186/s12967-022-03809-6)
Supplement: Supplementary file 5 — Additional file 5. Fig. S5: Barplots of the beta regression coefficients of the effect of the treatment (Metformin vs. Placebo) on the scaled metabolite changes identified in the main pooled analysis, estimated in the subgroup analysis including only the Italian sample (in blue) and only the USA sample (in dark yellow), for both targeted metabolomics (a) and untargeted metabolomics (b). [file 12967_2022_3809_MOESM5_ESM.docx]

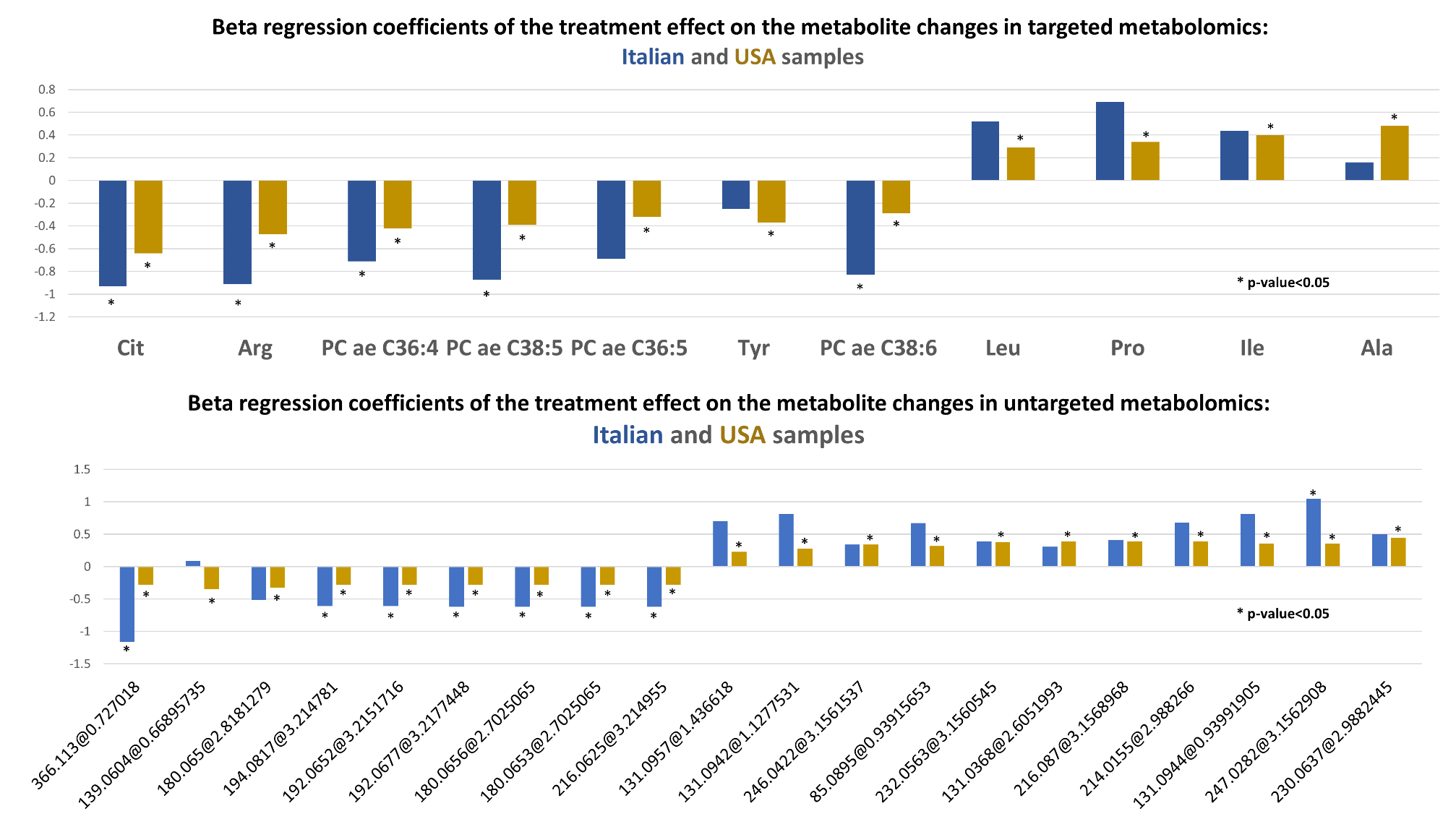


a.

b.

**Supplementary Figure S5.** Barplots of the beta regression coefficients of the effect of the treatment (Metformin vs Placebo) on the scaled metabolite changes identified in the main pooled analysis, estimated in the subgroup analysis including only the Italian sample (in blue) and only the USA sample (in dark yellow), for both targeted metabolomics (a) and untargeted metabolomics (b). A positive beta coefficient indicates a bigger increase in time of the metabolite in the metformin arm compared to placebo arm, whereas a negative coefficient indicates a bigger decrease of the metabolite in the metformin arm.

The multivariate regression models including only the Italian sample were adjusted for the scaled baseline value of the metabolite, menopausal status and change in BMI (body-mass index). The multivariate regression models including only the USA sample were adjusted for the scaled baseline value of the metabolite, menopausal status and change in BMI. BMI=body-mass index.

All the significant associations (p-value<0.05) between the treatment and the metabolite changes (in both targeted and untargeted metabolomics) in the USA subgroup analyses and in the Italian subgroup analyses are indicated with *.
