# Supplementary material for: Metabolomic profiles of metformin in breast cancer survivors: a pooled analysis of plasmas from two randomized placebo-controlled trials
Source: J Transl Med. 2022 Dec 29;20:629. doi: 10.1186/s12967-022-03809-6 (PMC9798585; doi:10.1186/s12967-022-03809-6)
Supplement: Supplementary file 6 — Additional file 6. Table S1: List of quantified metabolites using the AbsoluteIDQ p180 Biocrates kit, organized by chemical classes. [file 12967_2022_3809_MOESM6_ESM.docx]

**Supplementary Table S1.** List of quantified metabolites using the AbsoluteIDQ p180 Biocrates kit, organized by chemical classes.

| **Amino acids (21)** | | | |
| --- | --- | --- | --- |
| Ala | Alanine | Lys | Lysine |
| Arg | Arginine | Met | Methionine |
| Asn | Asparagine | Orn | Ornithine |
| Asp | Aspartate | Phe | Phenylalanine |
| Cit | Citrulline | Pro | Proline |
| Glu | Glutamate | Ser | Serine |
| Gln | Glutamine | Thr | Threonine |
| Gly | Glycine | Trp | Tryptophan |
| His | Histidine | Tyr | Tyrosine |
| Ile | Isoleucine | Val | Valine |
| Leu | Leucine |  | |

| **Biogenic amines (12)** | | | |
| --- | --- | --- | --- |
| alpha-AAA | alpha-Aminoadipic acid | Sarcosine | Sarcosine |
| ADMA | Asymmetric dimethylarginine | Serotonin | Serotonin |
| Creatinine | Creatinine | Spermidine | Spermidine |
| t4-OH-Pro | trans-4-Hydroxyproline | Spermine | Spermine |
| Kynurenine | Kynurenine | SDMA | Symmetric dimethylarginine |
| Putrescine | Putrescine | Taurine | Taurine |

| **Monosaccharides (1)** | |
| --- | --- |
| H1 | Hexoses (including glucose) |

| **Sphingomyelins (14)** | | | | |
| --- | --- | --- | --- | --- |
| SM (OH) C14:1 | | SM C18:0 | SM (OH) C22:2 | SM (OH) C24:1 |
| SM C16:0 | | SM C18:1 | SM C24:0 | SM C26:0 |
| SM C16:1 | | SM C20:2 | SM C24:1 | SM C26:1 |
| SM (OH) C16:1 | SM (OH) C22:1 | |  | |

| **Acylcarnitines (20)** | | | | |
| --- | --- | --- | --- | --- |
| C0 | Carnitine | | C12:1 | Dodecenoylcarnitine |
| C2 | Acetylcarnitine | | C14 | Tetradecanoylcarnitine |
| C3 | Propionylcarnitine | | C14:1 | Tetradecenoylcarnitine |
| C4 | Butyrylcarnitine | | C14:2 | Tetradecadienylcarnitine |
| C4-OH (C3-DC) | Hydroxybutyrylcarnitine (Malonylcarnitine) | | C16 | Hexadecanoylcarnitine |
| C5 | Valerylcarnitine | | C16:1 | Hexadecenoylcarnitine |
| C8 | Octanoylcarnitine | | C16:2 | Hexadecadienylcarnitine |
| C10 | Decanoylcarnitine | | C18 | Octadecanoylcarnitine |
| C10:1 | Decenoylcarnitine | | C18:1 | Octadecenoylcarnitine |
| C12 | | Dodecanoylcarnitine | C18:2 | Octadecadienylcarnitine |

| **Glycerophospholipids (77)** | | | |
| --- | --- | --- | --- |
| lysoPC a C16:0 | PC aa C36:1 | PC aa C42:4 | PC ae C38:5 |
| lysoPC a C16:1 | PC aa C36:2 | PC aa C42:5 | PC ae C38:6 |
| lysoPC a C17:0 | PC aa C36:3 | PC aa C42:6 | PC ae C40:1 |
| lysoPC a C18:0 | PC aa C36:4 | PC ae C30:0 | PC ae C40:2 |
| lysoPC a C18:1 | PC aa C36:5 | PC ae C32:1 | PC ae C40:3 |
| lysoPC a C18:2 | PC aa C36:6 | PC ae C32:2 | PC ae C40:4 |
| lysoPC a C20:3 | PC aa C38:0 | PC ae C34:0 | PC ae C40:5 |
| lysoPC a C20:4 | PC aa C38:3 | PC ae C34:1 | PC ae C40:6 |
| lysoPC a C28:1 | PC aa C38:4 | PC ae C34:2 | PC ae C42:1 |
| PC aa C28:1 | PC aa C38:5 | PC ae C34:3 | PC ae C42:2 |
| PC aa C30:0 | PC aa C38:6 | PC ae C36:0 | PC ae C42:3 |
| PC aa C32:0 | PC aa C40:1 | PC ae C36:1 | PC ae C42:4 |
| PC aa C32:1 | PC aa C40:2 | PC ae C36:2 | PC ae C42:5 |
| PC aa C32:2 | PC aa C40:3 | PC ae C36:3 | PC ae C44:3 |
| PC aa C32:3 | PC aa C40:4 | PC ae C36:4 | PC ae C44:4 |
| PC aa C34:1 | PC aa C40:5 | PC ae C36:5 | PC ae C44:5 |
| PC aa C34:2 | PC aa C40:6 | PC ae C38:0 | PC ae C44:6 |
| PC aa C34:3 | PC aa C42:0 | PC ae C38:2 |  |
| PC aa C34:4 | PC aa C42:1 | PC ae C38:3 |  |
| PC aa C36:0 | PC aa C42:2 | PC ae C38:4 |  |
