# Supplementary material for: Metabolomic profiles of metformin in breast cancer survivors: a pooled analysis of plasmas from two randomized placebo-controlled trials
Source: J Transl Med. 2022 Dec 29;20:629. doi: 10.1186/s12967-022-03809-6 (PMC9798585; doi:10.1186/s12967-022-03809-6)
Supplement: Supplementary file 7 — Additional file 7. Table S2: List of quantified fatty acids using gas chromatography. [file 12967_2022_3809_MOESM7_ESM.docx]

**Supplementary Table S2.** List of quantified fatty acids using gas chromatography.

| **Fatty acids (61) *** | | | |
| --- | --- | --- | --- |
| Percent 14:0 | Percent 18:2n-6tc | Percent 22:4n-6 | Desaturation Index P16:1n-7c-n-9c/P16:0 |
| Percent 14:1n-5 | Percent 18:2n-6tt | Percent 22:5n-3 | Desaturation Index P18:1n-9c/P18:0 |
| Percent 15:0 | Percent 18:3n-3 | Percent 22:5n-6 | P18:3n-6/P18:2n-6 |
| Percent 15:1 | Percent 18:3n-3ctt | Percent 22:6n-3 | P20:4n-6/P20:3n-6 |
| Percent 16:0 | Percent 18:3n-3tcc | Percent 24:0 | P22:6n-3/P20:5n-3 |
| Percent 16:1n-7c/n-9c | Percent 18:3n-6 | Percent 24:1n-9 | Trans total |
| Percent 16:1n-7t/n-9t | Percent 20:0 | Percent CLA 10t 12c | Trans ruminant |
| Percent 17:0 | Percent 20:1n-9c | Percent CLA 9t 11c | Trans total industrial |
| Percent 17:1 | Percent 20:2n-6c | Saturated fatty acids | Trans total industrial-c18 |
| Percent 18:0 | Percent 20:3n-3 | Monounsaturated fatty acids-cis | Long chain Pufa-W6 |
| Percent 18:1n-12/9/8t | Percent 20:3n-6 | Polyunsaturated fatty acids-3 | Long chain Pufa-W3 |
| Percent 18:1n-5c | Percent 20:3n-9 | Trans Pufa3 | Ratio W6/W3 |
| Percent 18:1n-7c | Percent 20:4n-6 | Polyunsaturated fatty acids-6 | Ratio long chain W6/W3 |
| Percent 18:1n-7t | Percent 20:5n-3 | Trans Pufa6 |  |
| Percent 18:1n-9c | Percent 22:0 | Trans Cla Pufa |  |
| Percent 18:2n-6 | Percent 22:1n-9 | Polyunsaturated fatty acids-9 |  |

* Nomeclature for x:y-z format: x is the number of carbons in the fatty acid, y the number of double bonds and z the position of the double bond. The letters c and t provide the isomer information (cis and trans, respectively).
