# Supplementary material for: Metabolomic profiles of metformin in breast cancer survivors: a pooled analysis of plasmas from two randomized placebo-controlled trials
Source: J Transl Med. 2022 Dec 29;20:629. doi: 10.1186/s12967-022-03809-6 (PMC9798585; doi:10.1186/s12967-022-03809-6)
Supplement: Supplementary file 8 — Additional file 8. Table S3: Participant baseline characteristics of the Italian and USA cohorts. [file 12967_2022_3809_MOESM8_ESM.docx]

**Supplementary Table S3.** Participant baseline characteristics of the Italian and USA cohorts.

|  | **Italian cohort**  **(N=40)** | **USA cohort**  **(N=333)** | ***P*-value*** |
| --- | --- | --- | --- |
| **Treatment, n (%)** |  |  |  |
| Placebo | 13 (32.5%) | 166 (49.8%) | .056 |
| Metformin | 27 (67.5%) | 167 (50.2%) |  |
| **Weight Loss intervention n (%)** |  |  |  |
| No | 40 (100%) | 167 (50.2%) | <.001 |
| Yes | 0 (0%) | 166 (49.8%) |  |
| **Age, median [Q1, Q3]** | 53.0 [47.7, 60.8] | 62.0 [57.0, 67.0] | <.001 |
| **BMI, median [Q1, Q3]** | 28.8 [26.8, 30.9] | 30.1 [27.5, 33.8] | .034 |
| **Menopausal status, n (%)** |  |  |  |
| Post-menopausal | 29 (72.5%) | 333 (100%) | <.001 |
| Pre-menopausal | 11 (27.5%) | 0 (0%) |  |
| **Pathologic Stage, n (%)** |  |  |  |
| Stage I | 16 (40.0%) | 161 (48.3%) | .764 |
| Stage II | 15 (37.5%) | 116 (34.8%) |  |
| Stage III | 7 (17.5%) | 56 (16.8%) |  |
| Missing | 2 (5.0%) | 0 (0%) |  |
| **Histology, n (%)** |  |  |  |
| Invasive Ductal Carcinoma | 37 (92.5%) | 247 (74.2%) | .017 |
| Invasive Lobular Carcinoma | 0 (0%) | 38 (11.4%) |  |
| Other/Unknown | 3 (7.5%) | 48 (14.4%) |  |
| **Tumor Grade, n (%)** |  |  |  |
| Grade I | 1 (2.5%) | 94 (28.2%) | <.001 |
| Grade II | 9 (22.5%) | 144 (43.2%) |  |
| Grade III | 26 (65.0%) | 89 (26.7%) |  |
| Unknown | 4 (10.0%) | 6 (1.8%) |  |
| **Estrogen Receptor Status, n (%)** |  |  |  |
| Negative | 34 (85.0%) | 49 (14.7%) | <.001 |
| Positive | 6 (15.0%) | 283 (85.0%) |  |
| Missing | 0 (0%) | 1 (0.3%) |  |
| **Progesterone Receptor Status, n (%)** |  |  |  |
| Negative | 35 (87.5%) | 77 (23.1%) | <.001 |
| Positive | 5 (12.5%) | 239 (71.8%) |  |
| Borderline/Not noted | 0 (0%) | 16 (4.8%) |  |
| Missing | 0 (0%) | 1 (0.3%) |  |
| **HER2 Status, n (%)** |  |  |  |
| Negative | 12 (30.0%) | 270 (81.1%) | <.001 |
| Positive | 24 (60.0%) | 51 (15.3%) |  |
| Not noted/Other | 0 (0%) | 9 (2.7%) |  |
| Missing | 4 (10.0%) | 3 (0.9%) |  |
| **Aromatase inhibitor therapy, n (%)** |  |  |  |
| No | 40 (100%) | 143 (42.9%) | <.001 |
| Yes | 0 (0%) | 190 (57.1%) |  |

**P-values* derived from Wilcoxon rank-sum test for numerical variables and from Chi-square test (or Fisher exact test, where appropriate) for categorical variables.
