# Supplementary material for: Metabolomic profiles of metformin in breast cancer survivors: a pooled analysis of plasmas from two randomized placebo-controlled trials
Source: J Transl Med. 2022 Dec 29;20:629. doi: 10.1186/s12967-022-03809-6 (PMC9798585; doi:10.1186/s12967-022-03809-6)
Supplement: Supplementary file 9 — Additional file 9. Table S4: The 20 features and the annotations achieved, providing the beta regression coefficients of the treatment covariate effect on feature changes in time and the corrected P-values. [file 12967_2022_3809_MOESM9_ESM.docx]

**Supplementary Table S4**. The 20 features and the annotations achieved, providing the beta regression coefficients of the treatment covariate effect on feature changes over time and the corrected *P*-values.

| **Annotated compound** | **Mass** | **Retention time (min)** | **Beta regression coefficient ^a^** | **FDR-corrected**  ***P*-value^b^** |
| --- | --- | --- | --- | --- |
| Caffeine ^c^ | 194.0817 | 3.21 | -0.31 | .031 |
| Caffeine | 192.0652 | 3.22 | -0.31 | .031 |
| Caffeine | 192.0677 | 3.22 | -0.31 | .031 |
| Caffeine (sodium adduct) | 216.0625 | 3.21 | -0.30 | .031 |
| Paraxanthine ^c^ | 180.0656 | 2.70 | -0.30 | .031 |
| Paraxanthine ^c^ | 180.0653 | 2.70 | -0.30 | .031 |
| Theophylline ^c^ | 180.065 | 2.82 | -0.33 | .029 |
| Isoleucine ^c^ | 131.0957 | 1.44 | 0.26 | .043 |
| Isoleucine/Leucine ^c,e^ | 131.0942 | 1.13 | 0.32 | .044 |
| Isoleucine/Leucine ^c,e^ | 85.0895 | 0.94 | 0.34 | .029 |
| Isoleucine/Leucine ^c,e^ | 131.0944 | 0.94 | 0.38 | .017 |
| 3-methyl-2-oxovalerate ^c,d^ | 214.0155 | 2.99 | 0.38 | .029 |
| 3-methyl-2-oxovalerate ^c,d^ | 230.0637 | 2.99 | 0.42 | .017 |
| 4-methyl-2-oxovalerate ^c,d^ | 216.087 | 3.16 | 0.38 | .029 |
| 4-methyl-2-oxovalerate ^c,d^ | 232.0563 | 3.16 | 0.37 | .029 |
| 4-methyl-2-oxovalerate ^c,d^ | 246.0422 | 3.16 | 0.33 | .044 |
| 4-methyl-2-oxovalerate ^c,d^ | 247.0282 | 3.16 | 0.40 | .027 |
| Indoxyl sulphate ^d^ | 131.0368 | 2.61 | 0.37 | .029 |
| Unknown | 366.113 | 0.73 | -0.36 | .028 |
| Unknown | 139.0604 | 0.67 | -0.34 | .031 |

^a^ Positive beta coefficient indicates a bigger increase in time of the metabolite in the metformin arm compared to placebo, whereas negative coefficient indicates a bigger decrease of the metabolite in the metformin arm.

^b^ *P*-value of the Treatment covariate (Metformin vs Placebo) derived from a multivariate linear model fit on scaled metabolite changes (final evaluation - baseline), adjusted for the scaled baseline value of the metabolite, study center, weight-loss intervention, age, change in BMI, ongoing aromatase-inhibitor therapy, histology, tumor grade, stage, HER2, progesterone receptor, estrogen receptor.

^c^ Compound identified at Level 1, using the corresponding analytical standard for confirmation of retention time and MS/MS fragmentation spectra ^3^.

^d^ Compound identified by analyzing the sample on negative mode.

^e^ Chromatographic artifact
